# Supplementary material for: Remote electrophysiological cardiotocography (eCTG), evaluation of feasibility in complicated pregnancies from 32 until 37 weeks gestational age in a home@hospital setting (HASTA): A prospective cohort study protocol
Source: PLoS One. 2026 Feb 3;21(2):e0341554. doi: 10.1371/journal.pone.0341554 (PMC12867231; doi:10.1371/journal.pone.0341554)
Supplement: S1 Appendix — (PDF) [file pone.0341554.s001.pdf]

Home-based **A**ntenatal surveillance with **S**elf-administered **T**elemonitoring **A**pproach

RESEARCH PROTOCOL HASTA study

Healthy **A**ging starts with a healthy **STA**rt

Remote electrophysiological cardiotocography (eCTG), evaluation of feasibility in (a selected group of) complicated pregnancies from 32 until 37 weeks gestational age in a Home@Hospital setting: a prospective cohort study.

Version 3.0 November 2024

PROTOCOL TITLE: HASTA- Healthy Aging starts with a healthy STArt

Remote electrophysiological cardiotocography (eCTG), evaluation of feasibility in (a selected group of) complicated pregnancies from 32 until 37 weeks gestational age in a Home@Hospital setting: a prospective cohort study

**Protocol ID**	NL87858.015.24
**Short title**	**HASTA**<br>Feasibility remote eCTG monitoring Home@Hospital
**Version**	3.0
**Date**	04-11-2024
**Coordinating investigator/project leader**	S. van Weelden, MSc, PhD candidate<br>Máxima MC<br>De Run 4600, 5504 DB, Veldhoven, The Netherlands<br>Email: s.vanweelden@mmc.nl<br>Phone (office): +31 40 8888384
**Principal investigator(s)**	dr. J.O.E.H. van Laar, gynaecologist-perinatologist, Signal Processing, Associate Professor, PhD<br>Máxima MC<br>De Run 4600, 5504 DB, Veldhoven, The Netherlands<br>Email: Judith.van.laar@mmc.nl<br>Phone (office): +31 408888384
**Fellow investigators**	dr.ir. M.B. van der Hout – van der Jagt, Msc, PhD, Medical Engineer, Assistant Professor<br>Máxima MC<br>De Run 4600, 5504 DB, Veldhoven, The Netherlands<br>Email: m.b.v.d.Hout@tue.nl<br>Phone (office): +31 40 8888384<br><br>dr. L. Monen, PhD, gynaecologist-fellow perinatology<br>Máxima MC<br>De Run 4600, 5504 DB, Veldhoven, The Netherlands<br>Email: loes.monen@mmc.nl<br>Phone (office): +31 40 8888384
**Statistical experts**	Prof. Dr. E.R. van den Heuvel, statistician<br>Eindhoven MedTech Innovation Center Mathematics and Computer Science, Statistics<br>Email: e.r.v.d.heuvel@tue.nl
**Sponsor**	Board of Management Máxima Medical Center
**Subsidising party**	Funded by University Fund Eindhoven, Nemo Healthcare, by Máxima Fund and by the PPP Allowance TKI HTSM,

	made available by Top Sector Holland High Tech to the University Fund Eindhoven to stimulate public–private partnerships, grant number PPS23-2-03529539.
**Independent expert (s)**	dr. H.J. Niemarkt<br>Pediatrician - Neonatologist<br>Máxima MC<br>De Run 4600, 5504 DB, Veldhoven, The Netherlands<br>Phone: 040 888 93 50

PROTOCOL SIGNATURE SHEET

Name	Signature	Date
**Coordinating Investigator:**<br>S. van Weelden, MSc, PhD candidate<br>Máxima MC<br>Email: s.vanweelden@mmc.nl<br>Phone (office): +31 40 8888384		04-11-2024
**Principal Investigator:**<br>Dr. J.O.E.H. van Laar, gynaecologist-Perinatologist, Signal Processing, Assistant Professor, PhD. Máxima MC<br>Email: Judith.van.laar@mmc.nl<br>Phone (office): +31 408888384		04-11-2024
**Fellow Investigators:**<br>dr.ir. M.B. van der Hout – van der Jagt, Msc, PhD, Medical Engineer, Assistant Professor<br>Máxima MC<br>De Run 4600, 5504 DB, Veldhoven, The Netherlands<br>Email: m.b.v.d.Hout@tue.nl<br>Phone (office): +31 40 8888384<br><br>Dr. L. Monen, PhD, gynaecologist-fellow perinatology<br>Máxima MC<br>De Run 4600, 5504 DB, Veldhoven, The Netherlands<br>Email: loes.monen@mmc.nl<br>Phone (office): +31 40 8888384		04-11-2024<br><br>04-11-2024

TABLE OF CONTENTS

1. INTRODUCTION AND RATIONALE 10
2. OBJECTIVES 12
  2.1 Primary objective 12
  2.2 Secondary objective(s) 12
  2.3 Additional objectives 12
3. STUDY DESIGN 13
4. STUDY POPULATION 14
  4.1 Population (base) 14
  4.2 Inclusion criteria 14
  4.3 Exclusion criteria 14
  4.4 Sample size calculation 16
5. TREATMENT OF SUBJECTS 17
  5.1 Investigational treatment 17
  5.2 Use of co-interventions 18
  5.3 Escape medication 18
6. INVESTIGATIONAL PRODUCT 19
  6.1 Name and description of investigational product(s) 19
  6.2 Summary of findings from non-clinical studies 20
  6.3 Summary of findings from clinical studies 20
  6.4 Summary of known and potential risks and benefits 20
    6.4.1 Clinical benefits 20
    6.4.2 Contraindications 21
    6.4.3 Safety 21
    6.4.4 Clinical warnings and potential risks 22
7. METHODS 24
  7.1 Study parameters/endpoints 24
    7.1.1 Main study parameter 24
    7.1.2 Secondary study parameters 24
    7.1.3 Additional parameters 28
    7.1.4 Baseline characteristics 28
  7.2 Randomisation, blinding and treatment allocation 30
  7.3 Study procedures 30
    7.3.1 Selection, counseling and inclusion 30
    7.3.2 Interventions 31
    7.3.3 Patient questionnaires 34
    7.3.4 Follow-up 34
    7.3.5 Data collection 35
    7.3.6 Healthcare professionals questionnaire 35
  7.4 Withdrawal of individual subjects 35
  7.5 Replacement of individual subjects after withdrawal 35
  7.6 Follow-up of subjects withdrawn from treatment 35

7.7 Premature termination of the study....35
8. SAFETY REPORTING....36
8.1 Temporary halt for reasons of subject safety....36
8.2 AEs, SAEs and SUSARs....36
8.2.1 Adverse events (AEs)....36
8.2.2 Serious adverse events (SAEs)....36
8.2.3 Follow-up of adverse events....37
8.2.4 Data Safety Monitoring Board (DSMB) / Safety Committee....37
9. STATISTICAL ANALYSIS....38
9.1 Primary study parameter(s)....38
9.2 Secondary study parameter(s)....38
9.2.1 The questionnaire data....38
9.2.2 Cost analysis....39
9.3 Additional measurements....39
9.4 Interim analysis (if applicable)....40
9.5 Missing data....40
10. ETHICAL CONSIDERATIONS....41
10.1 Regulation statement....41
10.2 Recruitment and consent....41
10.3 Objection by minors or incapacitated subjects (if applicable)....41
10.4 Benefits and risks assessment, group relatedness....41
10.5 Compensation for injury....41
10.6 Incentives (if applicable)....41
11. ADMINISTRATIVE ASPECTS, MONITORING AND PUBLICATION....42
11.1 Handling and storage of data and documents....42
11.2 Monitoring and Quality Assurance....42
11.3 Amendments....42
11.4 Annual progress report....43
11.5 Temporary halt and (prematurely) end of study report....43
11.6 Public disclosure and publication policy....43
12. STRUCTURED RISK ANALYSIS....44
12.1 Potential issues of concern....44
12.2 Synthesis....44
13. REFERENCES....45

LIST OF ABBREVIATIONS AND RELEVANT DEFINITIONS

**ABR**	General Assessment and Registration form (ABR form), the application form that is required for submission to the accredited Ethics Committee; in Dutch: Algemeen Beoordelings- en Registratieformulier (ABR-formulier)
**AE**	Adverse Event
**AR**	Adverse Reaction
**CA**	Competent Authority
**CCMO**	Central Committee on Research Involving Human Subjects; in Dutch: Centrale Commissie Mensgebonden Onderzoek
**CTG**	Cardiotocografy
**CV**	Curriculum Vitae
**DSMB**	Data Safety Monitoring Board
**D-QUEST**	Dutch version of the Quebec User Evaluation of Satisfaction with assistive Technology
**DU**	Doppler Ultrasound
**eCTG**	Electrophysiological cardiotocography
**EU**	European Union
**GCP**	Good Clinical Practice
**GDPR**	General Data Protection Regulation; in Dutch: Algemene Verordening Gegevensbescherming (AVG)
**HCP**	Healthcare professional
**IB**	Investigator's Brochure
**IC**	Informed Consent
**METC**	Medical research ethics committee (MREC); in Dutch: medisch-ethische toetsingscommissie (METC)
**NFMS**	Nemo Fetal Monioring System
**NI-fECG**	Non-invasive fetal electrophysiological cardiography
**NRM**	Nemo Remote® Monitoring
**PIH**	Pregnancy induced hypertension
**PPQS**	Patient participation and satisfaction questionnaire
**(S)AE**	(Serious) Adverse Event
**Sponsor**	The sponsor is the party that commissions the organisation or performance of the research, for example a pharmaceutical company, academic hospital, scientific organisation or investigator. A party that provides funding for a study but does not commission it is not regarded as the sponsor, but referred to as a subsidising party.
**TOCO**	Tocodynamometry
**UAVG**	Dutch Act on Implementation of the General Data Protection Regulation; in Dutch: Uitvoeringswet AVG
**US**	Ultrasound
**VAS**	Visual analogue scale
**WMO**	Medical Research Involving Human Subjects Act; in Dutch: Wet Medisch-wetenschappelijk Onderzoek met Mensen

SUMMARY

Rationale: Patients with obstetric complications like pre-eclampsia (PE), fetal growth restriction (FGR) or preterm pre-labor rupture of membranes (PPROM) often need hospitalization for fetal and maternal monitoring. Remote home monitoring has the potential to decrease the psychological and family burden of a hospital admission and to increase patient satisfaction while reducing health care costs due to a reduction in antenatal admissions. Previous research shows no indications that fetal home monitoring of selected complicated pregnancies imposes additional risks compared to in-hospital monitoring (1). Due to the limitations of conventional remote CTG monitoring like signal loss, electrophysiological cardiotocography (eCTG) has been developed and it is certified from 21 weeks of pregnancy onward. Since signal quality of eCTG in the preterm period – due to signal loss – is still unclear (2), the feasibility needs to be assessed first. The HASTA study will evaluate the feasibility of remote eCTG monitoring using non-invasive fetal electrocardiography (NI-fECG), Nemo Remote® Monitoring (NRM) in a hospital setting, as if the patient is at home (i.e., Home@Hospital setting). Automated interpretation of remote eCTG will be evaluated aiming to improve fetal monitoring and perinatal outcomes in the future. Additionally, this study will evaluate the feasibility of synchronized multimodal acquisitions of maternal and fetal measurements in a small population, aiming to understand the (patho)physiological mechanisms underlying cardiovascular function in pregnancy in the future.
Objective: To evaluate the feasibility of remote eCTG monitoring in complicated pregnancies between 32-37 weeks in a Home@Hospital setting. Secondary objectives are to evaluate automated interpretation of remote eCTG monitoring and to evaluate perinatal and maternal outcomes, patients and healthcare professionals satisfaction, and costs. Additional objective is to evaluate the feasibility of synchronized multimodal acquisitions of maternal and fetal measurements.
Study design: A single center interventional prospective cohort study.
Study population: Pregnant patients (N=60) >18 years old with a singleton, high-risk pregnancy from 32 until 37 weeks of gestational age. High-risk is defined as: diagnosed with PE, FGR, PPROM, or others with an indication for fetal monitoring at least twice a week, these patients are eligible for inclusion in this study. The main exclusion criteria are: suspected labor within 48 hours, severe pre-eclampsia, umbilical artery Doppler with absent- or reversed flow, external- or implanted electrical stimulator and language barrier.
Intervention: Remote eCTG monitoring (using Nemo Remote®), daily in a Home@Hospital setting for 30-90 minutes, or at least twice weekly at the outpatient clinic. Monitoring duration depends on signal quality and interpretability of the eCTG tracing, and lasts at least 30 minutes, and will be ceased after 90 minutes if the eCTG signal quality and/or interpretability is then still insufficient. In 24 patients ultrasound clips of 2 x 5 minutes of the uterine and umbilical artery Doppler will be collected simultaneously to the eCTG measurements.
Main study parameters/endpoints: Primary outcome: The percentage of successful eCTG measurements in a Home@Hospital setting. This will be defined as the necessity to switch to conventional CTG based on the amount of signal loss. Secondary outcomes: Automated interpretation of the eCTG, maternal and perinatal outcomes, patient and healthcare professional satisfaction and costs. Additional outcome: Feasibility of synchronized multimodal acquisitions of maternal and fetal measurements.

Nature and extent of the burden and risks associated with participation, benefit and group relatedness: Participation in this study is expected not to cause any risk for the patient or fetus. In case eCTG registration is insufficient, a switch to the conventional CTG can be made.
The benefits of eCTG monitoring with Nemo Remote® include the fact that it is wireless, non-invasive and well positionable without the help of elastic belts. As a result, repetitive repositioning to reduce signal loss - as required when using conventional CTG frequently performed with the support of healthcare professionals (HCPs) - is not needed. This is beneficial for future implementation of home monitoring throughout pregnancy, thereby increasing the patient's autonomy. Conducting self-administered remote eCTG home monitoring in a hospital setting, enables the evaluation of the feasibility at home - most likely - without any risk to patient or fetus. Patients using Nemo Remote® have a very small – known and unrelated to remote monitoring – probability of developing skin irritation or a minor (local) allergic reaction to the skin electrodes from the abdominal patch. There is no need for treatment if skin irritation happens, as this will naturally resolve over the course of days once the patch is removed (as in NIEM-O W22.070, NIEM-II W22.071). Measurements of the umbilical- and uterine artery are widely applied in routine obstetric care and are known not to cause any harm to patient and/or fetus (3). At three moments participants will be asked to fill in digital questionnaires. All other interventions are standard care. No additional – study-related – hospital visits or fetal registrations are needed.

1. INTRODUCTION AND RATIONALE

E-Health solutions
The use of digital health for remote monitoring in pregnancy care is increasingly popular (4). In complicated pregnancies, monitoring with cardiotocography (CTG) is recommended in international guidelines to assess maternal and fetal conditions resulting in recurrent outpatient visits or hospital admission (5-8). Antenatal admissions often pose psychological stress to pregnant women because of separation from family and home, lack of activity, and feelings of uncertainty (4). In general, digital health has the potential to improve access to care, thereby increasing patient satisfaction while reducing health care costs due to a reduction in visits and admissions (9, 10). Telemonitoring is a relatively new approach in complicated pregnancy and is recognized as an alternative to hospital admission (1, 9-11). Home monitoring in pregnancy can also attribute to the principal of family-integrated care (FIC) starting in pregnancy. Multiple telemonitoring platforms for remote CTG have been evaluated in prospective studies, and their feasibility and acceptability by patients and clinicians are proven (4). However, there are limitations in using conventional CTG equipment.

Limitations of conventional remote CTG
The conventional CTG signals that are generated with Doppler Ultrasound (DU) and tocodynamometry (TOCO) are prone to signal artefacts and signal loss due to fetal and maternal movements, particularly in patients with a high body mass index (BMI) (12, 13). With an estimated prevalence of obesity of at least 12% (and at most 30%) of adults in the Netherlands in 2024, this is a common issue (14, 15). As a result of poor signal quality, the transducer often has to be repetitively repositioned (16) which complicates self-administered home monitoring. Additionally, loss of signal might be stressful for patients and patients experience the tightly fitted elastic bands as uncomfortable and this substantially limits their mobility (16).

Remote electrophysiological cardiotocography monitoring (eCTG) – Nemo Remote® (NRM)
Due to the limitations of conventional CTG monitoring, extensive research has been carried out to develop new, non-invasive, monitoring methods. Remote eCTG – including NI-fECG – monitoring has shown to be a very promising technique (17-20). This technique uses a wireless and beltless electrode patch that is placed on the maternal abdomen (Figure 1). It monitors fetal heart rate (FHR) by fetal electrocardiography (fECG), maternal heartrate by maternal electrocardiography, and the electrical activity of the uterine muscle by electrohysterography (EHG) (21-24). The electrophysiological recordings are hardly affected by maternal or fetal movement or abdominal wall thickness, as they are transferred through conduction of electrical signals across the underlying tissues (13, 25). Furthermore, the wireless electrode patch on the maternal abdomen gives patients the ability to move (16). The HASTA study will evaluate the feasibility of self-administered home monitoring in a Home@Hospital setting for a selected groups of complicated pregnancies. Feasibility will be evaluated by assessing the success rate (percentage of successful measurements) of remote eCTG, and whether no switch to conventional CTG due to interpretability is needed. Success rate is defined as maximum of 20% signal loss of total recorded time or at least 30 minutes of sufficient CTG interpretability. For further description see sections 2.1 and 7.1.1.

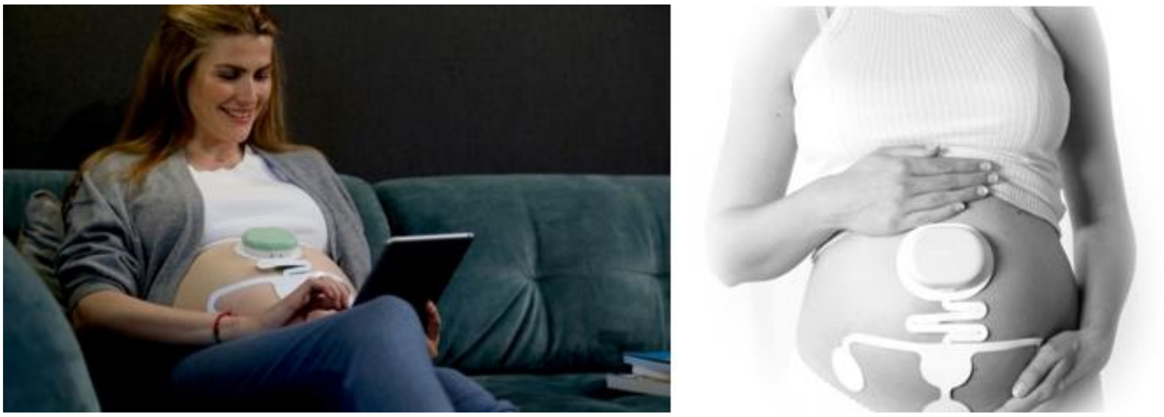

Figure 1. eCTG including NI-fECG – Nemo Remote® (NRM)

Furthermore, the FHR obtained from the fetal ECG measurements collected through NRM in this study will be used to check if the algorithm of the FHR – developed during labor – can also benefit for premature fetus. Automated interpretation of fetal monitoring could lead to improved prevention in the future. In addition, the effects of self-administered remote eCTG monitoring in a Home@Hospital setting on maternal and perinatal outcomes, patient and HCP satisfaction, and costs will be evaluated.

Besides feasibility of eCTG, this study will additionally evaluate the feasibility of synchronized multimodal acquisitions of maternal and fetal measurements as a first step towards the development of a prediction model.

2. OBJECTIVES

2.1 Primary objective

The primary objective of this study is to assess the percentage of successful self-administered remote eCTG (including NI-fECG) measurements in a Home@Hospital setting. This will be defined based on the percentage of signal loss and total duration of adequate registration and will be assessed on the first or – when indicated – the second eCTG measurement on the first day of inclusion. When the first remote eCTG measurement is reassuring but insufficient, the measurement will be repeated the same day.

2.2 Secondary objective(s)

The secondary objective is to evaluate the interpretability of eCTG measurements throughout the hospital period as described in section 2.1 and defined in section 7.1.1. In addition – regarding eCTG measurements – will be assessed the number of repeated eCTG measurement the same day – because of insufficient interpretability – including possible causes of poor signal quality, and the number of switches from eCTG to conventional CTG including possible reasons. Furthermore, will be assessed automated interpretation and the effects of self-administered remote eCTG monitoring in a Home@Hospital setting on maternal and perinatal outcomes, patient and HCP satisfaction, and costs.

2.3 Additional objectives

In addition, this study aims to evaluate the feasibility of synchronized multimodal acquisitions of maternal and fetal measurements to understand the (patho)physiological mechanisms underlying cardiovascular function in pregnancy. This is evaluated in a subset of 24 patients, where simultaneously with the eCTG measurements, Doppler US measurements of the umbilical and uterine arteries will be performed. In addition, automated interpretation of the – in this study collected – NI-fECG measurements through NRM will be evaluated, aiming to improve fetal monitoring, support the HCPs' interpretation and perinatal outcomes in the future.

3. STUDY DESIGN

The study will be conducted as a single center prospective cohort study between November 2024 and April 2026, aiming to include 60 eligible patients between 32 and 37 weeks of gestational age. The study is situated at the obstetric department of the Máxima Medical Center (MMC), a secondary and tertiary maternity care unit. However the aim of this study is to evaluate home monitoring in a hospital setting (Home@Hospital), only the use of remote eCTG through a NRM connection will differ from the standard care. A detailed description of the study population including sample size calculation, treatment of subjects, methods and statistical analysis, is provided in chapters 4,5,7 and 9. For study design and study procedures see Figure 2. The study will be registered on 'ToestingOnline' and 'www.clinicaltrials.gov'.

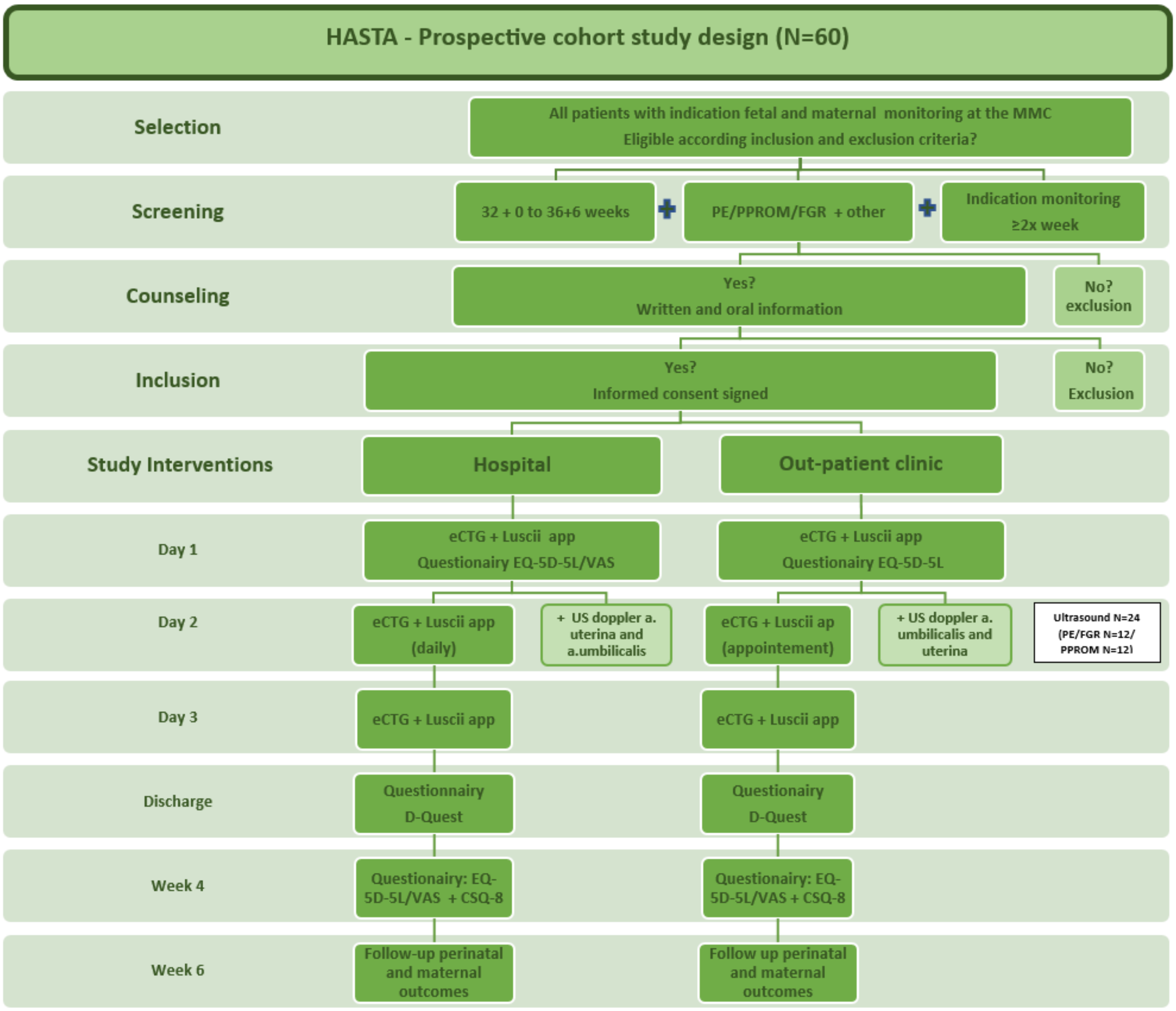

Figure 2. Flowchart of the HASTA prospective cohort design and study procedures.

4. STUDY POPULATION

4.1 Population (base)

In this prospective cohort study, eligible pregnant patients (N=60) will be included.
See sections 4.2 and 4.3 for inclusion and exclusion criteria, and Table 1 for definitions.

4.2 Inclusion criteria

In order to be eligible to participate in this study, a subject must meet all of the following criteria:

- Minimum age of 18 years old
- Pregnant patients with a gestational age between 32+0 and 36+6 weeks and days
- Singleton pregnancy
- Any indication for fetal monitoring at least twice per week (e.g.):
  - PE
  - FGR
  - PPROM
- Absence of exclusion criteria > 24 hours after admission.
- Oral and written informed consent is obtained.

4.3 Exclusion criteria

A potential subject who meets any of the following criteria will be excluded from participation in this study:

- An indication for intravenous medication
- Blood pressure >160/110mmHg
- Absent-/or reversed flow umbilical artery Doppler
- HELLP (Table 1)
- Obstetric intervention expected <48 hours, e.g. due to:
  - Non reassuring CTG
  - Active vaginal blood loss
  - Signs of abruption placentae
  - Meconium stained amniotic fluid
  - Signs of chorioamnionitis
- Patients admitted with a clinical diagnosis of sepsis with hypotension (i.e. septic shock).
- Insufficient knowledge of Dutch or English language
- Insufficient comprehension of instruction Nemo Remote® or patient information
- Fetal and/or maternal cardiac arrhythmias
- Contraindications to abdominal patch placement (dermatologic diseases of the abdomen precluding preparation of the abdomen with abrasive paper)
- Patients connected to an external or implanted electrical stimulator, such as Transcutaneous Electro Neuro Stimulation (TENS) and pacemaker (because of disturbance of the electrophysiological signal).

Table 1. Definitions of pregnancy related diagnosis

**Pre-eclampsia (PE)**
Pre-eclampsia is pregnancy induced hypertension (PIH) accompanied by one or more of the following new-onset conditions at or after 20 weeks' gestation (26):<br>1. Proteinuria<br>2. Other maternal organ dysfunction, including:<br>o Acute kidney injury (AKI) (creatinine ≥90 µmol/L; 1 mg/dL)<br>o Liver involvement (elevated transaminases e.g. ALT or AST > 40 IU/L) with or without right upper quadrant or epigastric abdominal pain)<br>o Neurological complications (examples include eclampsia, altered mental status, blindness, stroke, clonus, severe headaches, persistent visual scotomata)<br>o Haematological complications (thrombocytopenia – platelet count below 150,000/µL, DIC, hemolysis)<br>3. Uteroplacental dysfunction (such as fetal growth restriction, abnormal umbilical artery Doppler wave form analysis, or stillbirth).
**Fetal growth restriction (FGR)**
Fetuses with an estimated fetal weight (EFW) or abdominal circumference (FAC) that is less than the 10th percentile for gestational age (27).
**Preterm pre-labor rupture of membranes (PPROM)**
Rupture of membranes before gestational age of 37 weeks without contractions (11).
**HELLP syndrome** (**H**aemolysis **E**levated **L**iver enzymes and **L**ow **P**latelets)
The combination of all or some of haemolysis elevated liver enzymes and thrombocytopenia (26). Combination of symptoms that signifies a more serious manifestation of PE.<br>* Haemolysis (LDH ≥600 U/L, haptoglobin <0.2g/L AND<br>* Elevated liver enzymes (ASAT or ALAT >70U/L) AND<br>* Low platelets (<100*109/L).

4.4 Sample size calculation

In a previous cohort study for conventional remote CTG monitoring, 4% of all tracings needed repetition (562 out of 12,649) and 0.9% (N=111) of the CTGs had to be repeated in the hospital. The success rate at first trace was 95.5% and after repetition 99% (28) .

A success rate of 90% is considered clinically sufficient based on previous studies (28-30) and HCPs' opinion. Only the first day of remote eCTG measurements will be used for determination of the feasibility by assessing the signal quality and success. If the remote eCTG measurement is technically insufficient but reassuring – according to current clinical policy – it can be repeated once at the same day and still be considered successful. Calculated on 90% chance of success according Wilson standard error of proportions (CI95% > lower limit of 82.5%), 60 subjects are needed for inclusion in this cohort study.

5. TREATMENT OF SUBJECTS

At MMC, the current device used for conventional CTG monitoring is the Philips Avalon FM 30 (Philips Healthcare, Eindhoven, the Netherlands). The FHR is measured non-invasively by DU. The uterine activity and maternal heartrate are monitored by TOCO.

5.1 Investigational treatment

All patients will receive routine care following national guidelines and/or local protocols. Participants at the clinic will receive self-administered remote eCTG monitoring by NRM once a day (*Figure 1*). In case of follow-up at the outpatient clinic, participants will receive self-administered remote eCTG monitoring by NRM once per day or at least twice a week, depending on their care path. The realtime eCTG measurement will be instantly transferred digitally to the hospital electronic patient file (Chipsoft HiX) and assessed real-time on interpretability by the HCP. Monitoring will last between 30-90 minutes depending on the interpretability and reassurance of the remote eCTG measurement. Criteria defining a successful measurement are described in a standard operating procedure (SOP) and will be available for HCPs at the hospital to avoid prematurely closing of the eCTG measurement (see section 7.1.1). All HCPs involved in this study will receive training on the study protocol. The study procedure and statistical analysis are described in section 7.3.2, chapter 9 and in Figure 12.

Monitoring will be repeated the same day when reassuring but insufficiently interpretable, or upon request of the HCP in case of concerns about maternal and/or fetal condition. Additional detailed information of the NRM is described under investigational product (chapter 6). When remote eCTG interpretability is insufficient – based on clinical assessment of HCPs – a switch to conventional CTG monitoring will take place. In this study, HCPs will be a asked to define clearly if a switch to conventional CTG was made based on insufficient interpretability or because of concerns about fetal and/or maternal condition (section 7.1.2).

From the second day onward, synchronized US Doppler measurements (clips of 2 x 5 minutes) of the umbilical and uterine arteries will be performed simultaneously with the remote eCTG for 24 patients ([images removed]
Figure 3). Measurements of two groups - cardiovascular low (PPROM; N=12) versus cardiovascular high-risk (PE/FGR; N=12) - will be collected and analyzed to evaluate the feasibility of synchronized multimodal acquisitions of maternal and fetal measurements.

[images removed]

Figure 3. Measurement fetal and maternal heartrate, Doppler arteria umbilicalis, Doppler uterine artery and uterine activity.

During routine hospital care, admitted patients are asked daily for possible symptoms, and vital functions (e.g. heartrate, blood pressure, temperature, fluid balance) will be collected. During the study, the participants of this Home@Hospital study will measure their own vital functions, since this would also be the case when they were in a true Hospital@Home setting. Patients will therefore also register their vital functions and symptoms (e.g. PE-symptoms, abdominal pain, contractions, blood loss and fetal movements) during all remote ECTG measurements, this is done in the Luscii® app on

their telephone or tablet. The Luscii® app is widely used in the Netherlands and is already implemented in MMC as regular clinical care. For pregnancy, a separate function is available in Luscii® which will be used in the HASTA study (*Figure 4*). For a more detailed description of procedure interventions, see section 7.3.2.

To assess wellbeing and satisfaction, participants will receive questionnaires through email by Research Manager® at inclusion, discharge and 4 weeks postpartum (see description in sections 7.3.3 and 9.2.1). To assess HCP satisfaction, all HCPs involved in the HASTA study will receive a digital questionnaire – through mail by Research Manager® – once the last participants has delivered or has reached the GA of 37 weeks. Survey is based on a validated patient questionnaire (31) and suitable for HCPs after minimal adjustments. The validated survey is attached to appendix B. For further description and analysis of the questionnaires (see sections 7.3.6 and 9.2.1)

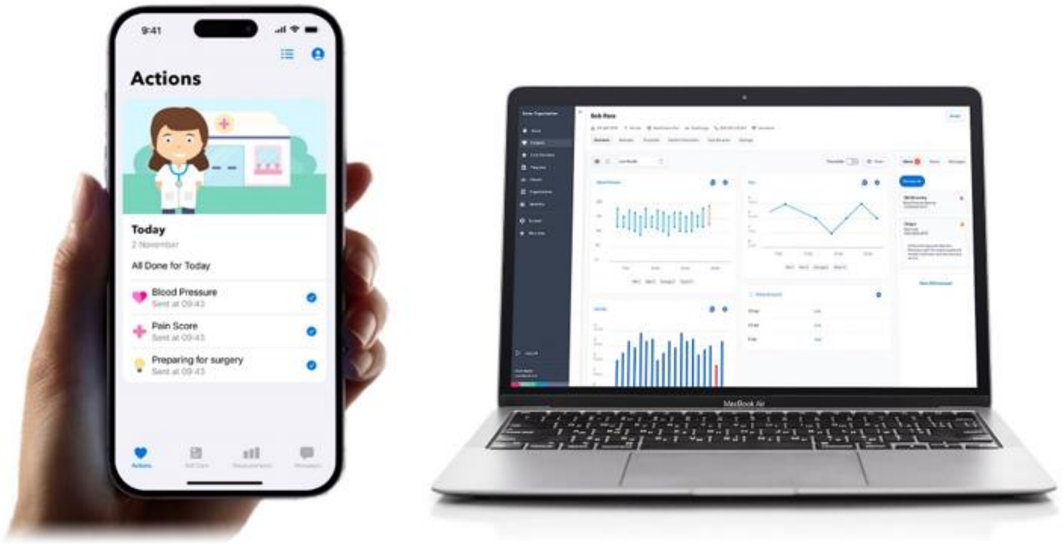

Figure 4. The Luscii app®

5.2 Use of co-interventions

Not applicable.

5.3 Escape medication

Not applicable.

6. INVESTIGATIONAL PRODUCT

6.1 Name and description of investigational product(s)

Nemo Remote® (NRM) NI-fECG Nemo Healthcare (v1.2.6)
https://a.storyblok.com/f/126579/x/afa5f256a9/nrm-techbro-eneu-v4-for-publishing.pdf
When available, the most recent release will be used in this study. Technical brochure NRM is available on request at Nemo Healthcare B.V.; https://nemohealthcare.com/en/contact/

Introduction and system identification

Nemo Remote (NRM) system is a non-invasive and wireless medical device that accurately monitors fetal and maternal heart rate and uterine activity. Nemo Remote components, accessories and compatible devices can be identified in the table below.

[removed for publication]
Figure 5. Nemo Remote® (NRM) system identification

Operation

Nemo Remote Link – a wearable electronic measurement unit, and Nemo Patch – a passive self-adhesive electrode sensor, are used for recording of electrophysiological signals from the maternal abdominal surface. These recordings contain a mixture of signals including maternal electrocardiogram (ECG), fetal ECG, electrohysterogram (EHG), and various noise sources. The NRM Link is battery operated and is charged by docking it to Nemo Remote Link Charger.
Nemo Remote App utilizes advanced signal processing software, installed on a tablet, to suppress noise sources and separate maternal ECG, fetal ECG, and EHG. The first two signals are used to calculate maternal heartrate and FHR, respectively. The latter is used to calculate a measure for uterine activity. Recorded data is transferred between NRM Link and the tablet via a wireless connection which utilizes low-power 2.4GHz ISM frequency band. The app also serves as the primary user interface.

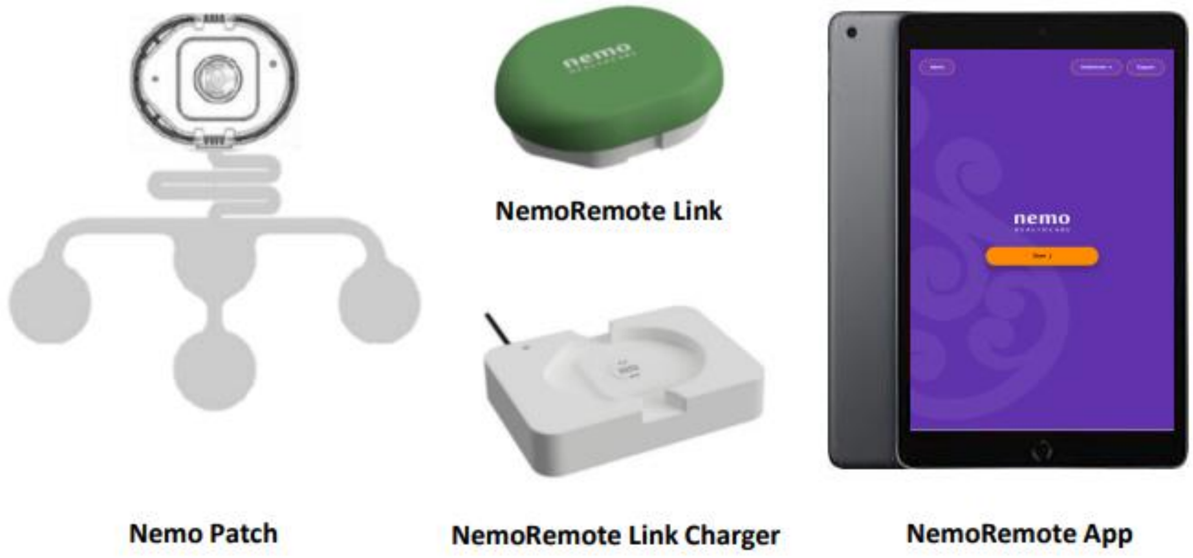

Figure 6. Nemo Remote® (NRM) patch, link, link charger and tablet app

Intended use
Nemo Remote® (NRM) is an electrophysiological measurement device that non-invasively measures FHR, uterine activity (UA) and maternal heart rate (MHR) from the fetal and maternal electrocardiography (ECG) and uterine electromyography (EMG) signals as acquired from abdominal surface electrodes. Nemo Remote® communicates to existing monitoring systems and is indicated for use on patients with a gestational age ≥ 21 completed weeks, with singleton pregnancies. The product is intended for use by patients in a home setting (prescribed by their HCP) and for use in non-acute care settings in a midwifery practice or hospital by HCPs.

6.2 Summary of findings from non-clinical studies

Not applicable.

6.3 Summary of findings from clinical studies

Not applicable.

6.4 Summary of known and potential risks and benefits

6.4.1 Clinical benefits

Direct benefits There are no benefits directly from the system itself.
Indirect benefits

- In comparison to current remote monitoring devices (based on DU) Nemo Remote® can also be used from 21 to 32 weeks of gestation.
- In comparison to fetal monitoring in the hospital Nemo Remote® provides the possibility to be monitored at home.

- In comparison to current non-invasive fetal monitoring techniques (DU + TOCO) Nemo Remote® has a non-inferior performance and decreased maternal discomfort.

6.4.2 Contraindications

The system is not intended for:
• Use in gestation period < 21 completed weeks
• Use in multiple pregnancies
• Use on patients with dermatologic diseases in which abdominal skin is involved
• Use on patients connected to external or implanted stimulators
• Use during defibrillation, electrosurgery or magnetic resonance imaging (MRI)
• Use in x-ray departments

6.4.3 Safety

NRM Link, NRM Link Charger and *NRM* App are parts of Nemo Remote® (NRM). Nemo Remote® is classified as class IIb, following Rules 10 and 11 of Chapter III of Annex VIII as well as implementation rules 3.3 and 3.5 of the Medical Device Regulation EU Regulation 2017/745.

Nemo Remote® complies with the following standards:
• EN 15223-1:2016
• EN ISO 20417:2021
• EN ISO 14971:2019
• IEC 60601-1:2005+AMD1:2012+AMD2:2020
• IEC 60601-1-2:2014+ AMD1:2020
• EN 60601-1-6: 2010+A1:2015
• IEC 60601-1-11:2015/AMD1:2020
• EN 62304:2006+A1:2015
• IEC 82304-1:2016
• EN 62366-1:2015+A1:2020
• Regulation (EU) No 207/2012

Nemo Patch is an accessory of Nemo Remote®, classified as Class I medical device according to rule 1 of Annex VIII of the Medical Device Regulation EU Regulation 2017/745.

The Nemo Patch complies with the following standards:
• EN 15223-1:2016
• EN 1041:2008 + A1:2013
• EN ISO 14971:2012
• EN 62366-1:2015
• EN ISO 10993-1:2018

6.4.4 Clinical warnings and potential risks

- The system is not a replacement for observation and evaluation of the patient and fetus at set times by a qualified healthcare provider, who makes diagnoses and takes decisions regarding treatment and interventions. For accurate care of the patient and fetus, clinical assessment of the CTG should be combined with knowledge of patient anamnesis and risk factors.
- The system is not designed for:
    - Use during defibrillation, electrosurgery or magnetic resonance imaging (MRI)
    - Use in x-ray departments.

  Remove the Patch prior to performing any of the above scenarios, otherwise harm to the patient or damage to equipment may occur.
- From 27 to 36 weeks gestational age, the chance of FHR signal loss is higher due to the presence of vernix caseosa, which can reduce transmission of electrical signals.
- In case clinical decision-making cannot be properly substantiated based on the CTG registration, other observation and monitoring methods must be used.
- In some cases, the use of the Patch, in combination with skin prep, may result in skin irritation. Try to reduce the duration of use and the skin prep as far as possible if the skin is irritated.
- To prevent reduced performance, replace a Patch when its adhesive properties have reduced or the electrode gel has come partially loose.
- To prevent cross-contamination, never reuse a Patch on another patient.
- To prevent the risk of skin irritation, ensure that no cleaning or disinfection agent residues remain on the system.
- The system detects repeating characteristics of the fetal and maternal ECG signal to calculate the fetal and maternal heart rate. If either the pregnant woman or her fetus has a highly irregular ECG, the heart rate may not be calculated correctly and the system may temporarily provide incorrect output. In case clinical decision-making cannot be sufficiently substantiated, other observation and monitoring methods must be used.
- In rare cases, the amplitude of the fetal ECG signal that is measured on the maternal abdomen is in the same order of magnitude as the amplitude of the maternal ECG that is being measured. In these circumstances, interchange of fetal and maternal heart rate may occur. The system will then temporarily display FHR values as maternal heart rate and maternal heart rate values as FHR. In case clinical decision-making cannot be sufficiently substantiated, other observation and monitoring methods must be used.
- The system is developed to detect FHR from extremely small fetal ECG signals in electrical measurements that may contain significant amounts of noise. The system evaluates the quality of the fetal ECG component in the measured signals. When the fetal ECG quality is too low to reliably detect the FHR, the FHR output is suppressed. In case clinical decision-making cannot be sufficiently substantiated, other observation and monitoring methods must be used.
- To prevent incorrect output, the system continuously evaluates the quality of the FHR output. If the FHR output repeatedly has not passed this quality check over a certain period of time, the FHR output is suppressed. In case clinical decision-making cannot be sufficiently substantiated, other observation and monitoring methods must be used.

- The system detects uterine activity based on electrohysterography (electrical activity of the uterine muscle). In limited cases, the registration may show deviations from the baseline that are not related to actual contractions, when compared to an intra-uterine pressure catheter. In case clinical decision-making cannot be sufficiently substantiated, other observation and monitoring methods must be used. Nemo Remote - Technical Brochure 7
- The system only provides qualitative information about uterine activity and no information about pressure.
- The measurement of abdominal fetal ECG, as performed by the system, is based on extremely small signals. For optimum measurement of these signals, the Link contains sensitive components that are subject to technological limitations. To prevent incorrect measurements through external electromagnetic interference, it is recommended to avoid using equipment that emits electromagnetic radiation in the vicinity of these measurements.
- The system shall only be used if the tablet is configured by mobile device management to ensure data privacy and guarantee the performance of the system.

[removed for publication]

Figure 7. Physical Specifications – Nemo Remote® Link and Link charger

[removed for publication]

Figure 8. Physical Specifications. Nemo Remote® App

[removed for publication]

Figure 9. Physical Specifications: Nemo Remote® accessories

[removed for publication]

Figure 10. Environmental Specifications

7. METHODS

7.1 Study parameters/endpoints

7.1.1 Main study parameter

To assess the primary objective of successful remote eCTG monitoring, definitions of successful measurement are as follows:

- Minimum of 30 minutes eCTG registration (32);
- Including overall a maximum of 20% signal loss (32);
- Sufficient interpretability according to HCPs clinical interpretation, if not maximum extended until 90 minutes when reassuring but insufficient.

Criteria defining a successful measurement is described in a standard operating procedure (SOP) and will be available for (trained) HCPs at the hospital to avoid prematurely closing of the eCTG registration. The precise duration of monitoring in minutes, the amount of signal loss in percentage and the duration of signal loss in minutes will be derived from the recordings during the analysis phase of the project, i.e. not during the measurements.

7.1.2 Secondary study parameters

Remote eCTG parameters

- Number of days with remote monitoring per participant.
- Number of remote eCTG measurements per participant.
- Need to repeat remote measurements the same day because of reassuring but insufficient interpretability.
- Number of switch to conventional CTG and possible maternal and/or fetal reason.
- Number of switch to conventional CTG needed because of poor signal of remote eCTG measurement and possible cause.
- Success rate of CTG measurement after switch based on same criteria as in remote eCTG monitoring (7.1.1).
- Incidence of skin irritation.
- Number of “unscheduled visits” and reason.
    - When there is an indication for an additional visit in-hospital as if the participant is monitored at home (e.g. contractions, decreased fetal movements, PE symptoms, suspicion of PPROM).
- Number of “re-admissions” and reason.
    - When there is an indication of admission as if the participant is monitored at home (e.g. blood loss, threatened premature labor, hypertension with indication for intravenous medication).
- The number of eCTG measurements assessed successful at closure by HCP.
- The number of eCTG measurements prematurely closed by HCP before complying the criteria of a successful measurement (7.1.1).

- Maternal and fetal factors HCPs have included in their assessment of the eCTG measurement (e.g. GA, fetal growth, Doppler measurements, PPROM).

Maternal parameters

- Gestational age at inclusion in days.
- Diagnosis and reason for monitoring.
- Duration from inclusion to delivery in days.
- Maternal morbidity
    - Emergency/secondary caesarean section + reason.
    - Postpartum hemorrhage, defined as >1000mL blood loss.
    - Abruptio placentae
    - Eclampsia
    - HELLP syndrome (Table 1)
    - Prolapse umbilical cord
    - Pulmonary- or deep venous thrombosis.
    - Chorionamnionitis
- Maternal mortality:
    - Defined as death from any cause related to or aggravated by the pregnancy or its management (excluding accidental or incidental causes) during pregnancy and childbirth or within 42 days of termination of pregnancy, irrespective of the duration and site of the pregnancy (33, 34)

Perinatal parameters

- Perinatal mortality
    - Defined as the number of fetal deaths past 22 completed weeks (154 days) of gestation plus the number of deaths among live-born children up to seven completed days of life (33, 34).

Neonatal parameters

- Gestational age at birth in days.
- Birth weight in grams and percentiles
- Admission and reason admittance medium care unit
- Admission and reason admittance neonatal intensive care unit
- Dysmaturity (<p3) or dysmaturity (<p10).
- Number of congenital anomalies.
- Asfyxia (Apgar score <7 at 5 minutes and/or umbilical cord pH <7.05).
- Apgar score <7 at 5 minutes.
- Umbilical cord pH <7.05.
- Neonatal mortality:
    - Defined as the number of neonatal deaths after the seventh day but before the 28th day of life (day 7-27) (33, 34).

Patient satisfaction parameters

To assess patient wellbeing and satisfaction, all included patients will receive the following validated (31) questionnaires through Research Manager®. At the first day of inclusion the EQ-5D-5L including VAS (31), before discharge the D-QUEST (35) and at 4 weeks after delivery EQ-5D-5L including VAS and CSQ-8 (31) questionnaires (7.3.3). The questionnaires can be digitally completed in on average 3-10 minutes. All surveys are attached as appendix (A, B and C). The analysis is described in section 9.2.1.

- At inclusion: EQ-5D-5L (36) including EQ VAS (visual analogue scale)**.** Questionnaire assessing the effect of the eCTG on patients health/wellbeing. The descriptive system comprises the following five dimensions: mobility, self-care, usual activities, pain/discomfort and anxiety/depression. Each dimension has five response levels: no problems, slight problems, moderate problems, severe problems, unable to /extreme problems. The participants are asked to indicate her health state by checking the box next to the most appropriate response level for each of the five dimensions. Responses are coded as single-digit numbers expressing the severity level selected in each dimension. The EQ VAS records the respondent's overall current health on a vertical visual analogue scale, where the endpoints are labelled 'The best health you can imagine' and 'The worst health you can imagine'. The EQ VAS provides a quantitative measure of the patient's perception of their overall health.
- Discharge: Satisfaction patient regarding the use of the eCTG D-QUEST (35) Dutch version of the Quebec User Evaluation of Satisfaction with assistive Technology.
- At 4 weeks after delivery: Satisfaction regarding the effect of the eCTG on their health/wellbeing EQ-5D-5L (36) including VAS (visual analogue scale) + satisfaction CSQ-8 (client/patient satisfaction questionnaire) (31).

Healthcare professional (HCP) satisfaction parameters

To assess HCP satisfaction concerning the eCTG, all HCP involved in the HASTA study will receive the following questionnaire questionnaire – through mail by Research Manager® – once the last participants has delivered or has reached the GA of 37 weeks.:

- D-QUEST (35) Dutch version of the Quebec User Evaluation of Satisfaction with assistive Technology.

Survey is based on a validated patient questionnaire (31) and suitable for HCP after minimal adjustments. An introduction will be given to clarify interpretation from HCP point of view. The validated survey is attached to appendix B and the analysis is described in section 9.2.1.

Costs supposing antenatal home monitoring versus in-hospital monitoring (ward, outpatient clinic).

To assess the costs of remote home monitoring versus in-hospital monitoring, patient journeys will be followed and costs calculated as if they were at home using remote eCTG compared to in- hospital using conventional CTG. The patient journey will not depend on participation in the HASTA study but is part of the standard care. Total of costs (mean and median) and the percentage of patients with at least one registered activity (% treated). In addition, a sub-analysis will be presented of participants needing hospitalization and of participants monitoring at the outpatient clinic at inclusion. Potential patient journey are shown in Figure 11.

Overview of cost analysis is presented in Table 2. The upper blue panel presents the population with indication to be monitored in-hospital. The patients' journey is presented by green circles (home monitoring) compared to the blue circles (in-hospital monitoring). The yellow circles in this panel presents the weekly scheduled visits at the outpatient clinic (standard care).

The lower yellow panel presents the population with indication to be monitored at the outpatient clinic. The patients' journey is presented by green circles (home monitoring), compared to the yellow circles (outpatient clinic monitoring) and/or blue circles (in-hospital monitoring). Every circle presents a day and the arrows presents the possible shifts during the patients' journey. For example, when hospitalization is indicated for a patient – initially monitored at the outpatient clinic – a shift to the blue line will take place. In some more exceptional cases, a patient – initially monitored in-hospital – can be discharged and will receive monitoring at the outpatient clinic. In that case, a shift to the yellow line will take place.

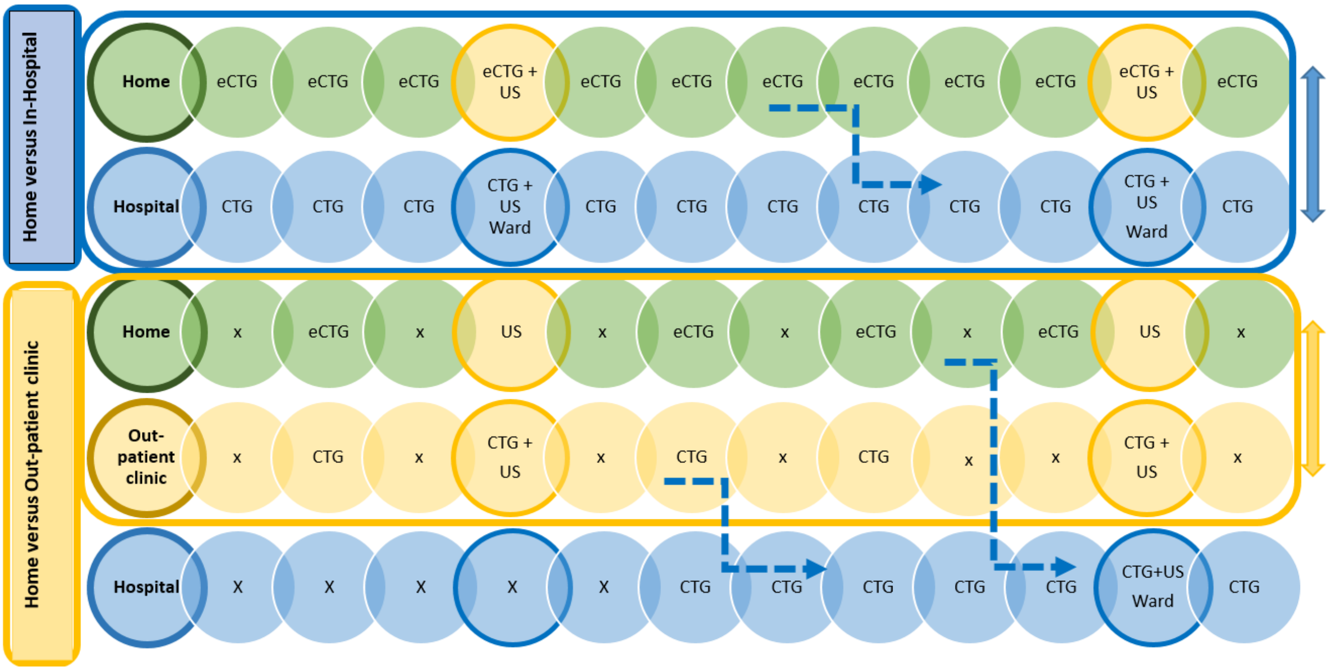

Figure 11. Patients Journey of Home versus In-Hospital monitoring (blue upper panel); Patients Journey Home versus Outpatient clinic monitoring (yellow lower panel).

<u>Potential cost components in a "Patients Journey Home monitoring" versus "In-Hospital admission":</u>

- Costs (euros) of Home admission.
  - Including total of consult(s) by telephone and use Luscii® app.
  - Based on total costs per patient (mean/median).
- Costs (euros) of remote eCTG during Home admission.
  - Based on costs total of eCTG measurements (30-90 minutes) per patient (mean/median).
- Costs (euros) of emergency ambulance transport (if needed).
  - Based on costs total of ambulance transport per patient (mean/median).
- Costs (euros) of unscheduled Outpatient clinic visits.

    - Based on costs total of additional visits (emergency) at the outpatient clinic per patient (mean/median).
- Costs (euros) of weekly scheduled Outpatient clinic visits.
    - Based on costs total of planned visits at the outpatient clinic once a week per patient (mean/median).
- Costs (euros) of the In-Hospital admission.
    - Based on costs total number of admission days per patient in case hospitalization is needed (mean/median).
- Costs (euros) of conventional CTG during In-Hospital admission.
    - Based on costs total number of conventional CTG measurements per patient in case hospitalization is needed (mean/median).

<u>Potential cost components in a "Patients Journey Home monitoring" versus "Outpatient clinic monitoring":</u>

- Costs (euros) of Home monitoring (≥ 2x/week).
    - Including total of consult(s) by telephone and use Luscii® app.
    - Based on total costs per patient (mean/median).
- Costs (euros) of remote eCTG during Home monitoring (≥ 2x/week).
    - Based on costs total of eCTG measurements (30-90 minutes) per patient (mean/median).
- Costs (euros) of emergency ambulance transport.
    - Based on costs total of ambulance transport per patient (mean/median).
- Costs (euros) of unscheduled outpatient clinic visits.
    - Based on costs total of additional visits (emergency) at the outpatient clinic per patient (mean/median).
- Costs (euros) of weekly scheduled outpatient clinic visits.
    - Based on costs total of planned visits at the outpatient clinic once a week per patient (mean/median).
- Costs (euros) of conventional CTG measurement (30-90 minutes) et the Outpatient clinic (≥ 2x/week).
    - Based on costs conventional CTG measurements at the Outpatient clinic per patient (mean/median).
- Costs (euros) of the In-Hospital admission.
    - Based on costs total number of admission days per patient in case hospitalization is needed (mean/median).
- Costs (euros) of conventional CTG during In-Hospital admission.
    - Based on costs number of conventional CTG measurements per patient in case hospitalization is needed (mean/median).

7.1.3 Additional parameters

Blood flow in the uterine artery and umbilical artery trough Doppler US (clips of 2 x 5 minutes). Raw data of the FHR trough eCTG monitoring. For statistical analysis of additional parameters see section 9.3.

7.1.4 Baseline characteristics

The following participant characteristics will be collected at baseline for descriptive analysis:

- Maternal age (in years).

- Ethnicity.
- Body mass index before pregnancy (BMI, kg/m2): < 30, >30, ≥ 40.
- Highest finished education:
    - Low, Middle, High, Missing.
- Parity: Nulliparous
- Medical history:
    - Chronic hypertension.
    - PIH.
    - Pre-existing diabetes mellitus.
    - Gestational diabetes mellitus.
    - Polycystic ovary syndrome (PCOS).
    - Thyroid disease.
    - Thrombophilia.
    - Uterine surgery.
    - Cardiac disease.
- Intoxications: smoking, alcohol use
- Previous spontaneous vaginal delivery.
- Previous caesarean delivery.
- Previous spontaneous preterm delivery (GA <37 weeks).
- Previous postpartum haemorrhage (>1000 milliliters blood loss).
- Gestational age at study entry (days, median/IQR).
- Diagnosis and reason for inclusion study:
    - PE
    - FGR
    - PPROM

      Other indication for fetal monitoring at least twice per week.

Additional maternal and neonatal data – in the first 6 weeks postpartum or at the point mother and/or neonate is discharged from the hospital will – be obtained from the electronic patient record and collected in the CRF.

Additional maternal data:

- Medical history: general history, family history, obstetric history
- Current pregnancy: prenatal diagnostic investigation and results, placental localization, obstetric complications during current pregnancy, medication use during current pregnancy
- Delivery: duration rupture of membranes, color and consistency of amniotic fluid, Group B Streptococcus (GBS) infection, start of labor, fetal position, complications during delivery (i.e. fever), mode of pain relief, mode of delivery (vaginal spontaneous, vacuum extraction, caesarean section, forceps), perineal tear, episiotomy, uterine rupture, blood transfusion.
- Complications postpartum: infection requiring antibiotics (urinary tract infection, chorioamnionitis, endometritis, infection following CS, pneumonia, mastitis).

Additional neonatal data:

- Diagnosis: intraventricular haemorrhage (IVH), periventricular leukomalacia (PVL), bronchopulmonary dysplasia (BPD), necrotizing enterocolitis (NEC), retinopathy of

prematurity (ROP), hypoxic ischemic encephalopathy, neonatal convulsions, neonatal sepsis (culture proven)
- Interventions: need for intubation, mechanical ventilation within the first 72 hours after birth, antibiotics within the first 72 hours after birth, spontaneous intestinal perforation (SIP) necessitating surgery, surfactant treatment.

7.2 Randomisation, blinding and treatment allocation
Not applicable.

7.3 Study procedures

7.3.1 Selection, counseling and inclusion
An overview of the study procedure is integrated in the flowchart presented in chapter 3.
Eligible patients are identified at admission or at the outpatient clinic if an indication for monitoring at least twice a week is set (1st day). All eligible patients will be consecutive asked if they agree to receive information about the study by the attending gynaecologist or resident. They will receive verbal and written information (patient information folder (PIF)) in Dutch or English about remote eCTG monitoring in a Home@Hospital setting. The information will be provided by another (trained) attending obstetric health care professional (resident, physician assistant (PA), (research) midwife or nurse). Possible further questions will be answered and counselling will be performed by one of the researchers or their delegates (trained and registered in a delegation log). Procedure as prescribed above, will ensure that patients participation will not be influenced by a dependent factor. Additionally, a brochure 'Wetenschappelijk onderzoek' will be assigned to the patient in 'mijnmmc' account – available for all patients – to provide general information about participation in scientific research. Patients will have 24 hours to decide if they want to participate in the study. After 24 hours (2nd day) – when still eligible – patient will provide informed consent and will receive a hard copy of the informed consent form. Given the nature of the study and the study population, it is necessary to deviate from standard Dutch regulations where a minimum reflection period of 7 days is required. This study concerns high-risk pregnant women where the delivery can take place within a short period of time (within a few days). In addition, to answer the research question, it is of great importance that fetal monitoring is examined early after diagnosis so the sample size of 60 participants will be achieved. Patients will be free to ask more time to think about it. When they meet the inclusion and exclusion criteria a few days after admission, it is still possible to participate in the study. Patients who are monitored on an outpatient basis have extra time to think about it until the next outpatient appointment if necessary.

A study number will be created in Research Manager® for every participant. After inclusion, the investigator or HCP will create a patient account through the general HASTA account on the Luscii® platform (see sections 2.2, 5.1). The participant will instantly receive through email an invitation to create a Luscii® account. Once the participant has downloaded the Luscii® app on their tablet or smartphone, users introduction will start and explain all functionalities. Participants without access to a tablet or smartphone will have the possibility to use the tablet of the NRM.
From the second day onwards, for each inclusion, it is assessed whether the participant fits the aimed profile (section 5.1) and whether the additional measurements are logistically feasible.

Participants will be asked if they agree to the additional measurements described in sections 2.3 and 7.1.3.

Patients who decide not to participate will receive standard care which consist in conventional CTG monitoring, measurements will be done by HCP and patients will not register their measurements and symptoms in the Luscii® app.

7.3.2 Interventions

Participants at the clinic will self-administer remote eCTG monitoring with the NRM (Figure 1) and will enter their self-performed measurements, symptoms and vital functions in the Luscii® app. For detailed description of the objectives and interventional treatment, see sections 2.1, 2.2 and 5.1. For an overview of the study interventions see Figure 12. Initially, patient' appointment at the outpatient clinic may be extended by 5 minutes due to the self-administered eCTG measurement, but ultimately the self-administered measurement probably will save time for the participants because there will be no waiting time for the HCP.

Participants will receive a Quick Start Guide (QSG) and for HCPs an Information For Users (IFU) manual of NRM will be available. Both manuals will be available at the ward and published on the local study website at the MMC. Documents will be added to the clinical trial management system (CTMS) in Research Manager® (NRM-QSG-NL_v2, NRM-IFU-NL v2).

Procedure Nemo Remote®, Luscii® app and additional interventions.

Participants who are hospitalized

- At inclusion the investigator or HCP will hand over a kit containing all material needed to start the self-administered eCTG (see section 6.1) and will go through all steps of the procedure together with the participants before they start the first self-administered eCTG measurement on which the primary outcome and sample size in this study is based. They will be asked to fill in their self-administered measurements, symptoms and vital functions on the Luscii® app. In addition, the participants will be asked to fill in a first digital questionnaire. A more detailed description of the questionnaires is defined in the following sections (2.2, 5.1, 7.1.2, 7.3.3 and 9.2.1).
- All subsequent days, in-hospital participants will perform daily their self-administered eCTG measurement as defined in sections 2.1 and 5.1 and procedure described in this section.
- In addition, from the second day onwards – synchronized US Doppler measurements of the umbilical and uterine arteries will be offered once to 24 participants with cardiovascular low- (PPROM) or cardiovascular high-risk (PE/FGR), and will be performed simultaneously with the remote eCTG (*[images* removed]
- *Figure 3*). Informed consent will be given at inclusion (11.1).
- Participants who are hospitalized at the ward will receive an appointment that will be planned in the digital (study specific) agenda (HiX).
- From the day of inclusion, the responsible HCP (PA) will sent a message through the Luscii® app to all participants the moment the remote eCTG can be started. The actual measurement is supposed to be taken within a pre-decided timeframe, in agreement with each participant.
- During the study, the participants of this study will measure their own vital functions (e.g. heartrate, blood pressure, temperature, fluid balance), like as if they were at home. Patients

will register their vital functions and symptoms (e.g. PE-symptoms, abdominal pain, contractions, blood loss and fetal movements) during all remote eCTG measurements in the Luscii® app on their tablet or smartphone (*Figure 4*).

- During the remote eCTG measurement, the responsible HCP (PA) will observe instantly the eCTG registration at the ward and will transfer the information from the Luscii® app to patient file in HiX.
- Once the eCTG measurement is considered successful – or when the maximum of 90 minutes has exceeded – the HCP will sent a message in the Luscii app® and will contact the participant by telephone a few minutes later, the time they have stopped the remote eCTG measurement. All findings will be discussed with the participant and completed in the patients' file in HiX. In addition, the HCP will visit the participant in the hospital room.
- When eCTG measurement is not successful but reassuring, the measurement will be repeated the same day on a moment agreed between participant and HCP (PA).
- When eCTG measurement is not interpretable after repetition on the first day of inclusion, eCTG will be defined not successful – primary outcome – and a switch to conventional CTG will take place. Equally to routine care, in- hospital care is needed and home- monitoring will not be possible.
- When after an additional clinical visit by the HCP (PA) "unscheduled consult" at participants room for other reasons than signal quality concerns (e.g. contractions, reduced fetal movements, patients concerns), without indication for hospitalization – eCTG adequate and reassuring – participants can continue "home monitoring" through eCTG like it will be once home monitoring is implemented in the future. No switch to conventional CTG will be needed. This additional visit will be recorded as part of the patient journey for later cost analysis purposes (Figure 11, Table 2).
- As long as remote eCTG measurements are successful, participants will continue the study interventions until discharge, delivery or when 37 weeks is reached. At any moment – when demanded by the participants or by the HCP – a switch to conventional CTG will be made.
- When reached a GA of 37 weeks, after delivery or at discharge, participants will be asked to fill in a second digital questionnaire (5.1, 7.3.3), and at 4 weeks after delivery a third questionnaire.

Participants with follow-up at the outpatient clinic

- At inclusion the investigator or HCP will hand over a kit containing all material needed to start the self-administered eCTG (see section 6.1) and will go through all steps of the procedure together with the participants before they start the first self-administered eCTG measurement on which the primary outcome and sample size in this study is based. They will be asked to fill in their self-administered measurements, symptoms and vital functions on the Luscii® app. In addition, the participants will be asked to fill in a first digital questionnaire. A more detailed description of the questionnaires is defined in the following sections (2.2, 5.1, 7.1.2, 7.3.3 and 9.2.1).
- All subsequent days, in-hospital participants will perform daily their self-administered eCTG measurement as defined in sections 2.1 and 5.1 and procedure described in this section.
- In addition, from the second day onwards – synchronized US Doppler measurements of the umbilical and uterine arteries will be offered to 24 participants with cardiovascular low-

(PPROM) or cardiovascular high-risk (PE/FGR), and will be performed simultaneously with the remote eCTG (*[images* removed]

- *Figure 3*). Informed consent will be given at inclusion (11.1).
- Participants with indication of follow-up at the outpatient clinic, will receive an appointment that will be planned in the digital (study specific) agenda (HiX) like it is actually done in routine hospital care. They will perform their measurements in a room at the outpatient clinic where the NRM suitcase, a blood pressure monitor and a thermometer will be available from the self-measurement kit. Participants at the outpatient clinic will be scheduled in the agenda of the PA. Outpatient clinic population will receive a document with instructions when to contact the hospital (e.g. blood loss, fever, PE symptoms, stained amniotic fluid).
- From the day of inclusion, the responsible HCP (PA) will sent a message through the Luscii® app to all participants the moment the NRM can be started. The actual measurement is supposed to be taken within a pre-decided timeframe, in agreement with each participant.
- During the study, the participants of this Home@Hospital study will measure their own vital functions (e.g. heartrate, blood pressure, temperature, fluid balance), like as if they were at home. Patients will register their vital functions and symptoms (e.g. PE-symptoms, abdominal pain, contractions, blood loss and fetal movements) during all remote eCTG measurements in the Luscii® app on their tablet or smartphone (*Figure 4*).
- During the remote eCTG registration, the responsible HCP will observe instantly the eCTG registration at the ward and will transfer the information from the Luscii® app to patient file in HiX.
- Once the eCTG measurement is considered successful – or when the maximum of 90 minutes has exceeded – the HCP will sent a message in the Luscii app® and will contact the participant by telephone a few minutes later, the time they have stopped the remote eCTG measurement. All findings will be discussed with the participant and completed in the patients' file in HiX.
- When eCTG registration is not successful but reassuring, the measurement will be repeated the same day on a moment agreed between participant and HCP.
- When eCTG is not interpretable after repetition on the first day of inclusion, eCTG will be defined not successful – primary outcome – and a switch to conventional CTG will take place. Equally to routine care, in- hospital care is needed and home- monitoring will not be possible.
- When after an "unscheduled consult" at the outpatient clinic for other reasons than signal quality concerns (e.g. contractions, reduced fetal movements, patients concerns), without indication for hospitalization – eCTG adequate and reassuring – participants can continue "home monitoring" through eCTG like it will be once home monitoring is implemented in the future. No switch to conventional CTG will be needed. The event will be recorded as part of the patient journey for later cost analysis purposes (Figure 11, Table 2).
- When participants – monitored at the outpatient clinic – are hospitalized for daily maternal and fetal monitoring, study interventions will continue as long as participants meet inclusion and exclusion criteria.
- As long as remote eCTG registrations are successful, participants will continue the study interventions until discharge, delivery or when 37 weeks is reached. At any moment – when demanded by the participants or by the HCP – a switch to conventional CTG will be made.

- When reached a GA of 37 weeks, after delivery or at discharge, participants will be asked to fill in a second digital questionnaire (5.1, 7.3.3), and at 4 weeks after delivery a third questionnaire.

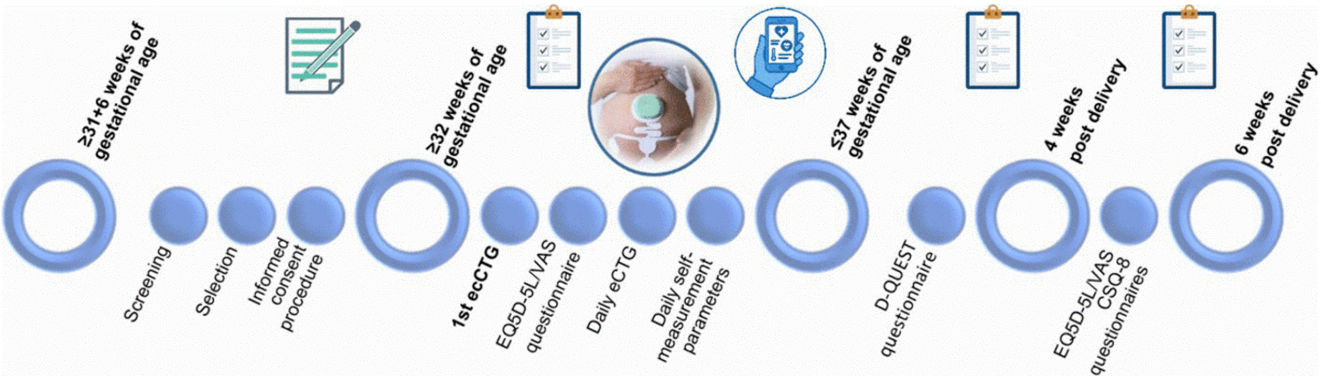

Figure 12. Overview study interventions: NRM - Luscii app - questionnaires.

7.3.3 Patient questionnaires

To assess patients wellbeing and satisfaction, all included patients will receive digital validated questionnaires on the first day of inclusion, before discharge and at 4 weeks after delivery in Research Manager® on their tablet of smartphone. All surveys are attached as appendix (A, B and C). For further description and analysis of the questionnaires see sections 7.1.2 and 9.2.1.

7.3.4 Follow-up

All patients will be followed up for six weeks to obtain neonatal and maternal outcomes. Data on maternal and neonatal mortality and morbidity will be noted during the routine postpartum check-up in the hospital or by their primary care midwife. Missing data will be requested from their primary care midwife, the child health clinic, the general physician, the pediatrician, and/or the gynecologist.

7.3.5 Data collection

At study entry, baseline data such as patient demographics, medical and obstetric history and current pregnancy details are collected. Relevant clinical data of mother and child during admission (e.g. gestational age, birth weight, Apgar scores, umbilical cord blood gas analysis) and until six weeks postpartum will be collected. Study parameters will be retrieved from the electronic patient record and questionnaires. Participants will give informed consent for the recruitment of their data until six weeks postpartum. The partner/legal representative who also has parental authority over the neonate will also be asked informed consent for the collection and the use of data of the neonate.

7.3.6 Healthcare professionals questionnaire

All HCPs involved in the HASTA study will receive a digital questionnaire – through mail by Research Manager® – once the last participants has delivered or has reached the GA of 37 weeks. Survey is based on a validated patient questionnaire (31) and suitable for HCPs after minimal adjustments. The validated survey is attached to appendix B. For further description and analysis of the questionnaires see section 9.2.1.

7.4 Withdrawal of individual subjects

Subjects can leave the study at any time for any reason if they wish to do so without any consequences. The investigators and HCPs can decide to withdraw or exclude a subject from the study when subject did not meet inclusion and exclusion criteria, when intervention was not received or for urgent medical reasons making measurements impossible or unsafe.

7.5 Replacement of individual subjects after withdrawal

When a subject withdraws from this study or does not meet the inclusion criteria – before start of the study interventions – no study number will be created and we will approach another patient for participation so that the requested sample size will be reached. An overview of inclusions, exclusions, refusals and missed eligible patients will be maintained in an “enrollment log” on the local server at the MMC, which will be protected by a password and has limited access by the healthcare providers directly involved.

7.6 Follow-up of subjects withdrawn from treatment

After withdrawal, there will be no follow-up of subjects.

7.7 Premature termination of the study

Not applicable.

8. SAFETY REPORTING

8.1 Temporary halt for reasons of subject safety

In accordance to section 10, subsection 4, of the WMO, the sponsor will suspend the study if there is sufficient ground that continuation of the study will jeopardise subject health or safety. The sponsor will notify the accredited METC without undue delay of a temporary halt including the reason for such an action. The study will be suspended pending a further positive decision by the accredited METC. The investigator will take care that all subjects are kept informed.

8.2 AEs, SAEs and SUSARs

8.2.1 Adverse events (AEs)

Adverse events are defined as any undesirable experience occurring to a subject during the study, whether or not considered related to the investigational product (e.g. skin irritation or minor (local) allergic reaction occurring within twelve hours after removal of the investigational product). All adverse events reported spontaneously by the subject or observed by the investigator or his staff will be recorded.

8.2.2 Serious adverse events (SAEs)

A serious adverse event is any untoward medical occurrence or effect that:

- Results in death;
- Is life threatening (at the time of the event);
- Requires hospitalization or prolongation of existing inpatients' hospitalization;
- Results in persistent or significant disability or incapacity;
- Is a congenital anomaly or birth defect; or
- Any other important medical event that did not result in any of the outcomes listed above due to medical or surgical intervention but could have been based upon appropriate judgement by the investigator.

An elective hospital admission will not be considered as a serious adverse event.
The investigator will report all SAEs, that occurred during hospitalization from application until twelve hours after removal of the investigational product, to the sponsor without undue delay after obtaining knowledge of the events:

- Maternal or fetal death for any reason;
- Severe allergic reaction of abdominal patch or application technique that requires hospitalization or prolongation of existing inpatients hospitalization;
- Any unforeseen important medical event as a result of the investigational product that needs medical intervention.

The investigator will report all SAEs to the sponsor without undue delay after obtaining knowledge of the events, except for the following SAEs:

- SAEs or AEs that did occur independently from the investigational product, but as a consequence of a (pregnancy or delivery related) disease, will not be reported to the sponsor. For example, criteria described by neonatal and maternal morbidity, shoulder dystocia, known congenital anomaly or birth defect, dysmaturity, macrosomia, hypoglycaemia, hyperbilirubinemia, feeding problem/weight loss, therapeutic hypothermia, perinatal asphyxia, observation for meconium-stained amniotic fluid, observation for suspected infection, because of maternal medication use in pregnancy, maternal intrapartum fever or maternal hypertensive disorders.
- The Investigator will report the SAEs through the web portal 'ToetsingOnline' to the accredited METC that approved the protocol, within 7 days of first knowledge for SAEs that result in death or are life threatening followed by a period of maximum of 8 days to complete the initial preliminary report. All other SAEs will be reported within a period of maximum 15 days after the sponsor has first knowledge of the serious adverse events.

8.2.3 Follow-up of adverse events

All AEs will be followed until they have abated, or until a stable situation has been reached. Depending on the event, follow up may require additional tests or medical procedures as indicated, and/or referral to the general physician or a medical specialist.
SAEs need to be reported till end of study within the Netherlands, as defined in the protocol.

8.2.4 Data Safety Monitoring Board (DSMB) / Safety Committee

Not applicable

9. STATISTICAL ANALYSIS

9.1 Primary study parameter(s)

Primary data statistical analyses will be performed using SPSS version 29 (IBM Corp., Armonk, NY, USA) and SAS software version 9.4 (SAS Institute Inc., Cary, NC, USA). Signal quality will be defined as the percentage of time (minutes) in which the remote eCTG device delivers an FHR value. Signal quality will be determined for each patient. Mean, SD, and 95% confidence interval (CI) of the mean success rate will be calculated using a one-sample t-test. The one-way ANOVA analog, Kruskal-Wallis test will be used in when no normal division of outcomes is found. Success rate will be defined as the number and percentage (N/%) of remote eCTG registration the first day of inclusion with at least 30 minutes interpretable signal quality and/or less than 20% signal loss during 30 to 90 minutes measurement (see section 7.1.1).

9.2 Secondary study parameter(s)

The generated data will be analyzed through SPSS (version 29). Baseline demographic, medical characteristics and secondary outcome measures of the included patients will be summarized. *If the* results are normally distributed, results will be reported as mean (standard deviation). If there is a non-normal distribution, results will be reported as median (interquartile ranges) or number (percentage). Several sensitivity analyses are being anticipated. Several subgroup analyses are investigated (e.g. PE, FGR, PPROM).

9.2.1 The questionnaire data

The questionnaires will be analyzed and will be summarized. The EQ-5D-5L questionnaire - including EQ VAS - 4 weeks after delivery will not be compared to the results at inclusion since no direct causality is expected. Where possible, the results of the EQ-5D-5L, EQ VAS and CSQ-8 questionnaires – where possible – will be descriptively compared to the outcomes of the HoTeL study (10).

- <u>EQ-5D-5L (36)</u>: Questionnaire assessing wellbeing. Summary statistics will be derived, including numbers of patients and proportions of categorical responses for the five EQ-5D dimensions. Results will be reported as mean (SD), minimum, median and maximum will be provided for change from inclusion to 4 weeks after delivery.
- <u>EQ VAS</u> (Visual Analogue Scale) - score between 0 and 100 - will be summarized. Results will be reported as mean (SD), minimum, median and maximum will be provided for change from inclusion to 4 weeks after delivery.
- <u>D-QUEST</u> (35): Questionnaire of 12 aspects that can influence the users' satisfaction of a device and the provision process. Results will be reported as not satisfied at all, displeased, more or less satisfied, satisfied or very satisfied.
- <u>CSQ-8</u> (31): Client/patient Satisfaction Questionnaire of 8 items including scores from 1 to 4. Items 2, 4, 5, and 8 are reverse scored. Total scores range from 8 to 32, with the higher number indicating greater satisfaction. Results will be reported as mean (SD), minimum and maximum.

9.2.2 Cost analysis

All health-care resources will be converted into cost estimates by multiplying the number of health-care units use by standard unit prices. The cost analysis will be performed by using a business case model. The economic analysis will estimate the value for money afforded by the remote eCTG monitoring Home monitoring compared to conventional In-Hospital CTG monitoring (ward/outpatient clinic). The cost of the antenatal care will be calculated following description in section 7.1.2 and Table 27.1.2. All results will be reported as mean, median and percentage (%). A sub-analysis will be presented of a home monitoring patients journey versus in-hospital and a home patients journey versus outpatient clinic patients journey.

Table 2: Cost analysis supposing antenatal Home monitoring versus In-Hospital monitoring (Ward, outpatient clinic) based on patients journey of the HASTA Home@Hospital population.

Antenatal care Costs	Home patients journey costs (N=60)			In-Hospital patients journey costs (N=60)			Difference
	Mean costs (€)	Median costs (€)	Treated (%)	Mean costs (€)	Median costs (€)	Treated (%)	Mean costs (€)
Home monitoring (admission day)				-	-	0%	
In-Hospital (admission day)							
Remote eCTG				-	-	0%	
Conventional CTG Outpatient clinic				-	-	0%	
Conventional CTG Ward							
Emergency transport				-	-	0%	
Visit outpatient clinic (scheduled)				-	-	0%	
Visit outpatient clinic (unscheduled/ emergency)				-	-	0%	
Total costs							

9.3 Additional measurements

Other study parameters - measurement fetal and maternal heartrate, Doppler umbilical and uterine artery ([images removed]
Figure 3) - will be analyzed by technical PhD students in collaboration with the TU/e with the aim to test the feasibility of these simultaneous measurements as a first step towards the development of a prediction model. The data collected through the additional measurements will be used to test if maternal-fetal coupling patterns change in cardio-vascular high- versus low-risk pregnancies. Also, the variability of the coupling parameters within the two groups will be evaluated. The Partial Directed Coherence (PDC) method will be used on simultaneously recorded fetal and maternal beat-by-beat heart rates collected from fetal and maternal eCTG signals, and on umbilical and uterine arteries parameters recorded from US signals of cardio-vascular high- (n=12) and low-risk (n=12)

pregnancies. The PDC fetal-to-maternal coupling analyses will provide information about the directionality and the magnitude of the cardiovascular coupling considering fetus-to-mother coupling (A_fBBI→ mBBI) and mother-to-fetus coupling (A_mBBI→ fBBI). Furthermore, this analysis will investigate how these coupling relationships can be mediated and influenced by the placenta (37).

The additional measurements are integrated into the HASTA protocol in the context of scientific collaboration between the MMC and the TU/e. As the HASTA study population exactly matches the population aimed for the measurements and analyses that the PhD students involved intend to perform. By integrating these measurements into the HASTA study, patients, healthcare personnel and the MMC research team are not unnecessarily burdened with a separate study for a few measurements. HASTA participants are informed in the PIF about the additional measurements and can choose to participate or not.

Measurement of FHR exported from the eCTG monitor collected in this study, will be analyzed by technical PhD student in collaboration with the TU/e – aiming to define the FHR profile of a healthy fetus and develop an automated interpretation of the fetal ECG measurement obtained from the eCTG measurements. The eCTG data collected in the HASTA study will be used to check if the algorithm of the FHR – developed during labor – can also benefit for premature fetus. If not, to define what steps are needed for further developments. Data will be analyzed by using a positive predictive metric for estimating sensitivity (Se), specificity (Sp) and positive predictive values (PPV). Results will be described in percentages (%) using Se, Sp, PPV (*p*-value) and their 95% confidence intervals (95% CI). All analyses will be done in collaboration with the clinical team, with the purpose to contribute to improve (future) pregnancy outcomes (section 11.6).

9.4 Interim analysis (if applicable)

Not applicable.

9.5 Missing data

Missing data will be assessed as number and percentage (N/%).

10. ETHICAL CONSIDERATIONS

10.1 Regulation statement

This study protocol and execution of the study will be fully compliant to the most recently updated version of the Declaration of Helsinki (64th version, 2013). Besides, it will be in accordance with the WMO. The local independent METC must give approval for the conduct of this study.

10.2 Recruitment and consent

A description is given in section 7.3.

10.3 Objection by minors or incapacitated subjects (if applicable)

Not applicable.

10.4 Benefits and risks assessment, group relatedness

Participation in this study is expected not to cause any risk for the patient or fetus. In case remote eCTG registration is insufficient, a switch to the conventional CTG can be made. The benefits of remote eCTG monitoring with Nemo Remote® include the fact that it is wireless (enabling patients to move), non-invasive and increases the autonomy of the patients. patients using the Nemo Remote® (NRM) have a very small probability of developing skin irritation or a minor (local) allergic reaction to the skin electrodes from the abdominal patch. There is no need for treatment if skin irritation happens (see NIEM-O W22.070, NIEM-II W22.071). Doppler measurements of the umbilical- and uterine artery is widely applied and is known not to cause any harm to patient and/or fetus (3). All other interventions are routine care.

10.5 Compensation for injury

Liability insurance policy has been obtained by MMC (policy number 626.107.173, Centramed). An insurance for subjects participating in this research is also available in accordance with the legal requirements of article 7 of the WMO (policy number 624.100.045, Centramed). This insurance provides cover for damage to research subjects through injury or death caused by the study. The insurance applies to the damage that becomes apparent during the study or within 4 years after the end of the study.

10.6 Incentives (if applicable)

Not applicable.

11. ADMINISTRATIVE ASPECTS, MONITORING AND PUBLICATION

11.1 Handling and storage of data and documents

We will do all effort needed to protect confidential personal data conform the rules and regulation stated in the Dutch General Data Protection Regulation (in Dutch: Wet Algemene Verordening Gegevensbescherming). Raw encrypted NRM data will be automatically stored at the Nemo Remote® (NRM) base. The researcher will transfer this data from the base to an external hard drive which is password-protected. Data stored at the base will be automatically deleted. The external hard drive will be securely stored at MMC in an enclosed space inside a secured office, and not leave MMC. The data will be pseudonymized and the decryption key will only be available in Máxima MC for members of the research team (all research members are mentioned on page 2 of this protocol). The raw encrypted NRM data will be sent to the protected network-attached storage at Nemo® Healthcare were the researchers can convert the data into the NRM parameters needed to answer the research questions. Nemo® Healthcare will not have access to the decryption key. The DICOM clips will be uploaded directly from the ultrasound to the password-protected external hard drive as described above. The files are pseudonymized. The decryption key will only be available in Máxima MC for members of the research team. All medical information as mentioned at paragraph 7.1.3 will be obtained from HiX at MMC or the medical files of subjects attending midwife. Research manager® (ISO 27001 certified) is used to build this database. We ask subjects for oral and written consent. All members of the research team will have access to Research Manager®. All data will be stored at MMC, location Veldhoven, for at least 15 years after completion of the study, this is a legal obligation.

11.2 Monitoring and Quality Assurance

Monitoring will be performed in compliance with Good Clinical Practice (GCP) to achieve high-quality research and secure patient safety. The Clinical Trial Center Maastricht will monitor the conduct of this study.

11.3 Amendments

Amendments are changes made to the research after a favourable opinion by the accredited METC has been given. All amendments will be notified to the METC that gave a favourable opinion.

A 'substantial amendment' is defined as an amendment to the terms of the METC application, or to the protocol or any other supporting documentation, that is likely to affect to a significant degree:

- The safety or physical or mental integrity of the subjects of the trial;
- The scientific value of the trial;
- The conduct or management of the trial; or
- The quality or safety of any intervention used in the trial.

All substantial amendments will be notified to the METC and to the competent authority. Non-substantial amendments will not be notified to the accredited METC and the competent authority, but will be recorded and filed by the sponsor.

11.4 Annual progress report

The sponsor/investigator will submit a summary of the progress of the trial to the accredited METC once a year. Information will be provided on the date of inclusion of the first subject, numbers of subjects included and numbers of subjects that have completed the trial, serious adverse events/ serious adverse reactions, other problems, and amendments.

11.5 Temporary halt and (prematurely) end of study report

The investigator/sponsor will notify the accredited METC of the end of the study within a period of 8 weeks. The end of the study is defined as the last patient's last visit 6 weeks postpartum and when questionnaire is completed. The sponsor will notify the METC immediately of a temporary halt of the study, including the reason of such an action. In case the study is ended prematurely, the sponsor will notify the accredited METC within 15 days, including the reasons for the premature termination. Within one year after the end of the study, the investigator/sponsor will submit a final study report with the results of the study, including any publications/abstracts of the study, to the accredited METC.

11.6 Public disclosure and publication policy

Conform the CCMO statement on publication policy, the positive as well as the negative results will be published. Results of this trial will be published at scientific meetings at (inter)national conferences and symposia and as publications in scientific journals. Articles will be written by the principal investigators, medical students and third parties participating in the study. Articles of the technical PhDs (students) from the TU/e, will be written in cooperation with the medical researchers from the MMC (section 9.3). Furthermore, anonymized open access data will be available to other researchers after publications of the article(s).

12. STRUCTURED RISK ANALYSIS

12.1 Potential issues of concern

a. Level of knowledge about mechanism of action

The NRM is implemented in Denmark and as implementation study in the Bravis hospital in Bergen op Zoom, The Netherlands. However, it has not been used in previous research, but remote eCTG with MONICA AN24 has recently been used in the study of Zizzo et al. (9).

- In Denmark, Nemo Healthcare proves that home monitoring is safe and efficient (tue.nl)
- Bravis start met thuismonitoring van ongeboren kind | Bravis

In addition, the eCTG has been used for previous and ongoing research projects, ID nr. NL57833.015.16, NL43294.015.13, NL48535.015.14, METC number 1407 and NL 68439.015.19. There is enough knowledge about the fECG and there appears to be no known pathophysiological consequences for the mother and the fetus. Currently NI-fECG is studied in the NIEM-O and NIEM-II study at the MMC (NL82869.015.22, NL82822.015.22).

b. Validation of Nemo Remote® (NRM) and patch

The Nemo Remote® (NRM) has a CE-mark and is validated in 2017 (>21wks) and introduced in 2022. The NRM is used for intended use only. Therefore, the NRM can be used during the antenatal period.

c. Safety assurance

To be sure that the safety of mother and child is guaranteed, a switch to the conventional CTG can be made at any time by the responsible clinician. If registration of FHR and/or EHG is insufficient without options for optimization of the signal, it can be decided to switch to the conventional CTG, which is available at the obstetric ward or outpatient clinic. This will not endanger mother and child

NRM Link, NRM Link Charger and NRM App are parts of Nemo Remote® (NRM). Nemo Remote® is classified as class IIb, following Rules 10 and 11 of Chapter III of Annex VIII as well as implementation rules 3.3 and 3.5 of the Medical Device Regulation EU Regulation 2017/745.
Nemo Patch is an accessory of Nemo Remote®, classified as Class I medical device according to rule 1 of Annex VIII of the Medical Device Regulation EU Regulation 2017/745.

12.2 Synthesis

Risk analyses are performed by the Clinical Physical Department of the Máxima MC Veldhoven. Before the study will start, the equipment will be tested by the Medical Technical Service Department, the Clinical Physical Department, Nemo Healthcare® and personnel from the obstetric ward (medical doctors, PAs and nurses) of MMC to guarantee safety.

13. REFERENCES

1. Li S, Yang Q, Niu S, Liu Y. Effectiveness of Remote Fetal Monitoring on Maternal-Fetal Outcomes: Systematic Review and Meta-Analysis. JMIR Publications Inc.; 2023.
2. Mannella P, Billeci L, Giannini A, Canu A, Pancetti F, Simoncini T, Varanini M. A feasibility study on non-invasive fetal ECG to evaluate prenatal autonomic nervous system activity. European Journal of Obstetrics & Gynecology and Reproductive Biology. 2020;246.
3. Alfirevic Z, Stampalija T, Dowswell T. Fetal and umbilical Doppler ultrasound in high-risk pregnancies. Cochrane Database of Systematic Reviews. 2017(6).
4. Van Den Heuvel JFM, Groenhof TK, Veerbeek JHW, Van Solinge WW, Lely AT, Franx A, Bekker MN. eHealth as the Next-Generation Perinatal Care: An Overview of the Literature. Journal of Medical Internet Research. 2018;20(6).
5. Royal College of Obstetricians and Gynaecologists. Small-for-Gestational-Age Fetus, Investigation and Management (Green-top Guideline No. 31). 2014.
6. National Institute of Clinical Excellence (NICE). Preterm labour and birth - NICE guideline [NG25]. 2022.
7. National Institute of Clinical Excellence (NICE). Hypertension in pregnancy: diagnosis and management - Clinical guidline [NG133]. 2023.
8. National Institute of Clinical Excellence (NICE). Antenatal care for uncomplicated pregnancies - Clinical guideline (2021).
9. Zizzo AR, Hvidman L, Salvig JD, Holst L, Kyng M, Petersen OB. Home management by remote self-monitoring in intermediate- and high-risk pregnancies: A retrospective study of 400 consecutive women. Acta Obstetricia et Gynecologica Scandinavica. 2021;101(1).
10. Bekker MN, Koster MPH, Keusters WR, Ganzevoort W, De Haan-Jebbink JM, Deurloo KL, et al. Home telemonitoring versus hospital care in complicated pregnancies in the Netherlands: a randomised, controlled non-inferiority trial (HoTeL). Lancet Digit Health. 2023(5(3):e116-e124.).
11. Van Den Heuvel JFM, Ganzevoort W, De Haan-Jebbink JM, Van Der Ham DP, Deurloo KL, Seeber L, et al. HOspital care versus TELemonitoring in high-risk pregnancy (HOTEL): study protocol for a multicentre non-inferiority randomised controlled trial. BMJ Open. 2019.
12. Pello L, Bernard N, Foch M-C-G, France Tr Caldeyro-Barcia S, Van Geijn H, Verloskunde A, et al. International Federation of Gynaecology 8~ Obstetrics FIG0 News GUIDELINES FOR THE USE OF FETAL MONITORING* Based upon the Workshop held in Zurich, Switzerland, March 28-29, 1985 by the FIG0 Subcommittee on Standards in Perinatal Medicine (Chairman: Goesta Rooth) Organised by GOESTA ROOTH**, Visiting Professor, ALBERT HUCH and RENATE HUCH, Professors.
13. Lempersz C, Noben L, Van Osta G, Wassen MLH, Meershoek BPJ, Bakker P, et al. Intrapartum non-invasive electrophysiological monitoring: A prospective observational study. Wiley; 2020. p. 1387.
14. Rodríguez-Mesa N, Robles-Benayas P, Rodríguez-López Y, Pérez-Fernández EM, Cobo-Cuenca AI. Influence of Body Mass Index on Gestation and Delivery in Nulliparous Women: A Cohort Study. MDPI AG; 2019.
15. Bemelmans Wje HRTVTLSVWMMSAJ. Toekomstige ontwikkelingen in matig overgewicht en obesitas. Inschatting effecten op de volksgezondheid. 2004.
16. Thijssen K, Vlemminx M, Westerhuis M, Dieleman J, Van Der Hout-Van Der Jagt M, Oei S. Uterine Monitoring Techniques from Patients' and Users' Perspectives. Georg Thieme Verlag KG; 2018. p. e184.
17. Euliano TY, Darmanjian S, Nguyen MT, Busowski JD, Euliano N, Gregg AR. Monitoring Fetal Heart Rate during Labor: A Comparison of Three Methods. Journal of Pregnancy. 2017.
18. Cohen WR, Ommani S, Hassan S, Mirza FG, Solomon M, Brown R, et al. Accuracy and reliability of fetal heart rate monitoring using maternal abdominal surface electrodes. Acta Obstetricia et Gynecologica Scandinavica. 2012;91(11).

19. Vlemminx MWC, Thijssen KMJ, Bajlekov GI, Dieleman JP, Van Der Hout-Van Der Jagt MB, Oei SG. Electrohysterography for uterine monitoring during term labour compared to external tocodynamometry and intra-uterine pressure catheter. Eur J Obstet Gynaecol and Repr Biol. 2017;215:197-205.
20. Reinhard J, Hayes-Gill BR, Schiermeier S, Hatzmann W, Herrmann E, Heinrich TM, Louwen F. Intrapartum signal quality with external fetal heart rate monitoring: a two way trial of external Doppler CTG ultrasound and the abdominal fetal electrocardiogram. Archives of Gynecology and Obstetrics. 2012;286(5).
21. Nederlandse Vereniging voor Obstetrie en Gynaecologie (NVOG) Richtlijn Intrapartum foetale bewaking à terme. [guideline fetal monitoring at term]. In Dutch. 2018;1–42.
22. Saccone G, Schuit E, Amer-Wåhlin I, Xodo S, Berghella V. Electrocardiogram ST Analysis During Labor. Obstetrics & Gynecology. 2016;127(1).
23. Oudijk MA, Kwee A, Visser GHA, Blad S, Meijboom EJ, Rosén KG. The effects of intrapartum hypoxia on the fetal QT interval. BJOG: An International Journal of Obstetrics & Gynaecology. 2004;111(7).
24. Behar J, Andreotti F, Zaunseder S, Oster J, Clifford GD. A practical guide to non-invasive foetal electrocardiogram extraction and analysis. Physiol Meas. 2016;37(5):R1-R35.
25. Cohen WR, Hayes-gill B. Influence of maternal body mass index on accuracy and reliability of external fetal monitoring techniques. Wiley; 2014. p. 590.
26. Brown MA, Magee LA, Kenny LC, Karumanchi SA, McCarthy FP, Saito S, et al. The hypertensive disorders of pregnancy: ISSHP classification, diagnosis & management recommendations for international practice. Pregnancy Hypertension. 2018;13.
27. Galan H, Grobman W. ACOG PRACTICE BULLETIN Clinical Management Guidelines for Obstetrician-Gynecologists. 2019.
28. Quemere MP, Boutroy JL, Fresson J, Abel F, Barbarino-Monnier P. Analyse de 12649 tracés.
29. Frenken MWE, Van der woude DAA, Vullings R, Oei SG, Van laar J. Implementation of the combined use of non-invasive fetal electrocardiography and electrohysterography during labor: A prospective clinical study. Acta Obstetricia et Gynecologica Scandinavica. 2023;102(7).
30. Kerner R, Yogev Y, Belkin A, Ben-Haroush A, Zeevi B, Hod M. Maternal self-administered fetal heart rate monitoring and transmission from home in high-risk pregnancies. Wiley; 2003. p. 33.
31. Larsen DL, Attkisson CC, Hargreaves WA, Nguyen TD, Nguyen TD. Assessment of client/patient satisfaction: Development of a general scale. Evaluation and program planning. 1979;2(3):197-207.
32. Fuchs T, Tomiałowicz M, Zimmer M. Signal Loss During Fetal Heart Rate Monitoring Using Maternal Abdominal Surface Electrodes Between 28 and 42 Weeks of Pregnancy. Adv Clin Exp Med. 2014;64(8):813-9.
33. World Health Organization. International statistical classification of diseases and related health problems, 10th revision, Fifth edition (2016).
34. De Sonnaville CMW, Hukkelhoven CW, Vlemmix F, Groen H, Schutte JM, Mol BW, Van Pampus MG. Impact of Hypertension and Preeclampsia Intervention Trial At Near Term-I (HYPITAT-I) on obstetric management and outcome in The Netherlands. Ultrasound in Obstetrics & Gynecology. 2019;55(1).
35. Wessels RD, Witte L. Reliability and validity of the Dutch version of QUEST 2.0 with users of various types of assistive devices. Disability and rehabilitation. 2003;25(6):267-72.
36. Versteegh MM, Vermeulen KM, Evers S, De Wit GA, Prenger R, Stolk EA. Dutch Tariff for the Five-Level Version of EQ-5D. Value in Health. 2016;19(4).
37. Khandoker AH, Schulz S, Al-Angari HM, Voss A, Kimura Y. Alterations in Maternal-Fetal Heart Rate Coupling Strength and Directions in Abnormal Fetuses. Front Physiol. 2019;10(Apr 26):482.

APPENDIX A

EQ5D5L (35) including EQ VAS (visual analogue scale)

Under each heading, please tick the ONE box that best describes your health TODAY.

MOBILITY

I have no problems in walking about ❑
I have slight problems in walking about ❑
I have moderate problems in walking about ❑
I have severe problems in walking about ❑
I am unable to walk about ❑

SELF-CARE

I have no problems washing or dressing myself ❑
I have slight problems washing or dressing myself ❑
I have moderate problems washing or dressing myself ❑
I have severe problems washing or dressing myself ❑
I am unable to wash or dress myself ❑

USUAL ACTIVITIES (*e.g. work, study, housework, family or leisure activities*)

I have no problems doing my usual activities ❑
I have slight problems doing my usual activities ❑
I have moderate problems doing my usual activities ❑
I have severe problems doing my usual activities ❑
I am unable to do my usual activities ❑

PAIN / DISCOMFORT

I have no pain or discomfort ❑
I have slight pain or discomfort ❑
I have moderate pain or discomfort ❑
I have severe pain or discomfort ❑
I have extreme pain or discomfort ❑

ANXIETY / DEPRESSION

I am not anxious or depressed ❑
I am slightly anxious or depressed ❑
I am moderately anxious or depressed ❑
I am severely anxious or depressed ❑
I am extremely anxious or depressed ❑

SAMPLE

- We would like to know how good or bad your health is TODAY.
- This scale is numbered from 0 to 100.
- 100 means the best health you can imagine.
  0 means the worst health you can imagine.
- Mark an X on the scale to indicate how your health is TODAY.
- Now, please write the number you marked on the scale in the box below.

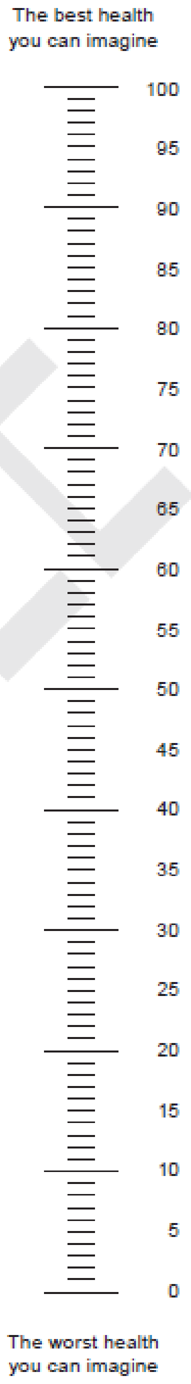

YOUR HEALTH TODAY =

SAMPLE

APPENDIX B

D-QUEST questionnaire

iRv, versie februari 2000

Naam cliënt: ....

Hulpmiddel / voorziening: ....

Datum: ....

Tevredenheid over uw hulpmiddel en de bijbehorende dienstverlening

Het doel van deze vragenlijst is na te gaan hoe tevreden u bent over uw hulpmiddel en de bijbehorende dienstverlening.

- Wilt u bij elk van de vragen aangeven hoe tevreden u bent over uw hulpmiddel en de bijbehorende dienstverlening, met behulp van de volgende 5 antwoord-mogelijkheden:

  Totaal niet tevreden
  Niet tevreden
  Min of meer tevreden
  Tevreden
  Zeer tevreden

- Wilt u alstublieft voor elk van de volgende vragen het antwoord aankruisen dat het best bij uw mate van tevredenheid past?
- Wilt u alstublieft alle vragen beantwoorden?
- Indien u niet helemaal tevreden bent, wilt u dan alstublieft de reden daarvan toelichten in de daarvoor bestemde ruimte achter de vraag?

1 Hoe tevreden bent u over de **afmetingen** van uw hulpmiddel? (maat, hoogte, lengte, breedte)	Totaal niet tevreden<br>Niet tevreden<br>Min of meer tevreden<br>Tevreden<br>Zeer tevreden	Reden ontevredenheid:
2 Hoe tevreden bent u over het **gewicht** van uw hulpmiddel?	Totaal niet tevreden<br>Niet tevreden<br>Min of meer tevreden<br>Tevreden<br>Zeer tevreden	Reden ontevredenheid:
3 Hoe tevreden bent u over de **verstel-mogelijkheden** van uw hulpmiddel?	Totaal niet tevreden<br>Niet tevreden<br>Min of meer tevreden<br>Tevreden<br>Zeer tevreden	Reden ontevredenheid:
4 Hoe tevreden bent u over de **veiligheid** van uw hulpmiddel?	Totaal niet tevreden<br>Niet tevreden<br>Min of meer tevreden<br>Tevreden<br>Zeer tevreden	Reden ontevredenheid:
5 Hoe tevreden bent u over de **duurzaamheid** van uw hulpmiddel? (bestendigheid, slijtvastheid)	Totaal niet tevreden<br>Niet tevreden<br>Min of meer tevreden<br>Tevreden<br>Zeer tevreden	Reden ontevredenheid:
6 Hoe tevreden bent u over het **gemak** waarmee u uw hulpmiddel kunt **gebruiken**?	Totaal niet tevreden<br>Niet tevreden<br>Min of meer tevreden<br>Tevreden<br>Zeer tevreden	Reden ontevredenheid:
7 Hoe tevreden bent u over het **comfort** van uw hulpmiddel?	Totaal niet tevreden<br>Niet tevreden<br>Min of meer tevreden<br>Tevreden<br>Zeer tevreden	Reden ontevredenheid:
8 Hoe tevreden bent u over de **effectiviteit** van uw hulpmiddel? (De mate waarin het hulpmiddel doet waarvoor het bedoeld is)	Totaal niet tevreden<br>Niet tevreden<br>Min of meer tevreden<br>Tevreden<br>Zeer tevreden	Reden ontevredenheid:

Hoe tevreden bent u, **alles bij elkaar genomen**, over uw **hulpmiddel?**	Totaal niet tevreden<br>Niet tevreden<br>Min of meer tevreden<br>Tevreden<br>Zeer tevreden	

9 Hoe tevreden bent u over het **verstrekkingsproces** waarmee u uw hulpmiddel heeft verkregen?<br>(procedures, tijdsduur)	Totaal niet tevreden<br>Niet tevreden<br>Min of meer tevreden<br>Tevreden<br>Zeer tevreden	Reden ontevredenheid:
10 Hoe tevreden bent u over de geboden **reparaties en onderhoud** voor uw hulpmiddel?	Totaal niet tevreden<br>Niet tevreden<br>Min of meer tevreden<br>Tevreden<br>Zeer tevreden	Reden ontevredenheid:
11 Hoe tevreden bent u over de **professionaliteit van de dienstverlening?**<br>(kwaliteit van de informatie en vakkundigheid van de dienstverleners)	Totaal niet tevreden<br>Niet tevreden<br>Min of meer tevreden<br>Tevreden<br>Zeer tevreden	Reden ontevredenheid:
12 Hoe tevreden bent u over de **service en dienstverlening na aflevering** van uw hulpmiddel?<br>(na-zorg, blijvende ondersteuning, begeleiding)	Totaal niet tevreden<br>Niet tevreden<br>Min of meer tevreden<br>Tevreden<br>Zeer tevreden	Reden ontevredenheid:

Hoe tevreden bent u, **al deze vier onderwerpen bij elkaar genomen**, over de **totale dienstverlening?**	Totaal niet tevreden<br>Niet tevreden<br>Min of meer tevreden<br>Tevreden<br>Zeer tevreden	

Hieronder is een lijst van dezelfde12 onderwerpen weergegeven.
Wilt u de drie onderwerpen die u het meest belangrijk vindt uitkiezen?

Zet een kruisje voor de **drie onderwerpen** die u het belangrijkste vindt.

Afmetingen	Comfort
Gewicht	Effectiviteit
Verstel-mogelijkheden	Verstrekkingsproces
Veiligheid	Reparaties en onderhoud
Duurzaamheid	Professionaliteit van de dienstverlening
Gebruiksgemak	Service en dienstverlening na aflevering

APPENDIX C

CSQ-8 (client/patient satisfaction questionnaire) (37).

(CSQ-8, v. TMS-180S) (Larsen et al., 1979) Permission received

Instructions for participants:

Please help us improve our service by answering some questions about the help that you have received. We are interested in your honest opinions, whether they are positive or negative. Please answer all of the questions. We also welcome your comments and suggestions. Thank you very much. We appreciate your help.

1. How would you rate the quality of service you received?

- Excellent (4)
- Good (3)
- Fair (2)
- Poor (1)

2. Did you get the kind of service you wanted?

- No, definitely not (1)
- No, not really (2)
- Yes, generally (3)
- Yes, definitely (4)

3. To what extent has our service met your needs?

- Almost all of my needs have been met (4)
- Most of my needs have been met (3)
- Only a few of my needs have been met (2)
- None of my needs have been met (1)

4. If a friend were in need of similar help, would you recommend our service to him or her?

- No, definitely not (1)
- No, I don't think so (2)
- Yes, I think so (3)
- Yes, definitely (4)

5. How satisfied are you with the amount of help you received?

- Quite dissatisfied (1)
- Indifferent or mildly dissatisfied (2)
- Mostly satisfied (3)
- Very satisfied (4)

6. Have the services you received helped you to deal more effectively with your problems?

- Yes, they helped a great deal (4)
- Yes, they helped somewhat (3)
- No, they really didn't help (2)
- No, they seemed to make things worse (1)

7. In an overall, general sense, how satisfied are you with the service you received?

- Very satisfied (4)
- Mostly satisfied (3)
- Indifferent of mildly dissatisfied (2)
- Quite dissatisfied (1)

8. If you were to seek help again, would you come back to our service?

- No, definitely not (1)
- No, I don't think so (2)
- Yes, I think so (3)
- Yes, definitely (4)

Scoring:

Scores are summed across items once.
Items 2, 4, 5, and 8 are reverse scored.
Total scores range from 8 to 32, with the higher number indicating greater satisfaction.
