# Supplementary material for: Remote electrophysiological cardiotocography (eCTG), evaluation of feasibility in complicated pregnancies from 32 until 37 weeks gestational age in a home@hospital setting (HASTA): A prospective cohort study protocol
Source: PLoS One. 2026 Feb 3;21(2):e0341554. doi: 10.1371/journal.pone.0341554 (PMC12867231; doi:10.1371/journal.pone.0341554)
Supplement: S4 Appendix — (PDF) [file pone.0341554.s004.pdf]

Subject information for participants in medical research

In-hospital data collection on the feasibility of home monitoring of mother and child during pregnancy up to 6 weeks post-delivery.

Official title: Evaluation of the feasibility of remote electrophysiological maternal and fetal monitoring in a home@hospital setting during pregnancy up to 6 weeks post-delivery.

Dear Madam,

You are receiving this letter because you are pregnant and your attending physician at the Máxima MC (MMC) has told you that it is important to check you and your child extra carefully — during admission or at the outpatient clinic. Through this information letter we want to inform you and ask if you want to participate in medical-scientific research. Participation is voluntary. To participate, your written permission is required.

Before you decide whether you want to participate in the study, you will receive an explanation of what the study entails. Here you can read what kind of study it is, what it means to you, and what the advantages and disadvantages are. It is a lot of information. Would you like to read the information and decide whether you want to participate? If you would like to participate, please fill out the form that can be found in Annex D. Please indicate if you need extra time to decide whether you want to participate.

Ask your questions

You can decide whether you want to participate based on the information provided in this information letter. In addition, we recommend that you do this:

- Ask questions to the researcher from whom you have received this information.
- Talk to your partner, family or friends about this study.
- Ask questions to the independent expert. For contact details see Appendix A .
- Read the information on *www.rijksoverheid.nl/mensenonderzoek.*

Further general information about participating in a medical scientific study can be found in your *mijnmmc* account where the Folder 'Scientific research' has been added on *www.mijn.mmc.nl*.

1. General information

The MMC has set up this study. Researchers carry out the study in MMC at the obstetric department or the outpatient clinic. These researchers can be doctors, physician assistants (PA), interns and healthcare workers. This study requires 60 subjects who are admitted to the obstetric ward or who visit the gynecology outpatient clinic of the MMC at least 2 times a week for extra check-ups. The medical ethics review committee Máxima Medical Center in Veldhoven has approved this study. The study is funded by the MMC, Eindhoven University of Technology (TU/e) and Nemo Healthcare®.

2. What is the background of the study?

If there is a reason during pregnancy to monitor the condition of mother and child more often, the pregnant woman will be admitted to the ward or asked to come to the outpatient clinic more often for extra check-ups. During admission to the hospital (or outpatient visits), a cardiotocography (CTG) or heart film is made as standard care.

Cardiotocography (CTG)

The CTG is intended to monitor the heart rate of mother and child and the activity of the uterus (contractions). This is done by placing two buttons on the abdomen. Two elastic bands around the waist keep these buttons in place. This way of monitoring has been used as standard care for decades (Figure 1). Sometimes it can be difficult to get a good registration of the heartbeat and contractions by using this method, partly because the movements of mother and child during registration. To improve the registration of the contractions, the contraction button must then be replaced every time. This can result in less freedom of movement and longer measurements due to signal loss.

Electrophysiological cardiotocography (eCTG)

In collaboration with the MMC, a new method has been developed – the Nemo Remote Monitor® (NRM) – to register mother and child remotely. This device registers the electrical signals emitted by the mother's heart, the child's heart, and the muscle of the uterus. These electrical signals are picked up by means of 1 patch on the mother's abdomen. The patch has a transmitter that transmits the signals wirelessly to the base station (Figure 2). Because the patch cannot move, the pregnant woman can move more easily. It is also expected that there will be less signal loss, so that the registration will have to be renewed less often. In recent years, a lot of research has been done into this new way of monitoring and it is already being used in some hospitals in regular care for home monitoring,

3. What is the purpose of the study?

With the regular CTG device, there is a greater risk of signal loss, which is why home monitoring during pregnancy is not widely used yet. With this study, we will look (in the hospital) whether home monitoring with the eCTG during pregnancy by using the NRM is more feasible. We do this by looking at how well the registration can be assessed. In addition, we assess at how often there is reason to switch to the CTG method. Our goal is to explore and assess the safety implications of home monitoring in the future. Hopefully, pregnant women will be able to stay at home more often in the future as an alternative to outpatient monitoring or even hospitalization, which is expected to lead to more satisfaction among the pregnant woman and her family.

[image removed for publication]

Figure 1. CTG

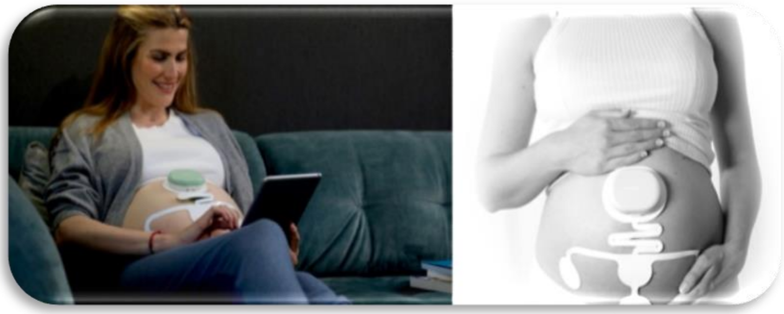

Figure 2. eCTG (NRM)

4. How does the study work?

How long does the study take?

Are you participating in the study? Then your participation will last as long as you are admitted to the obstetric department or as long as you come to the outpatient clinic for extra check-ups for you and your child. Up to 6 weeks after delivery, information about pregnancy and postpartum is collected and stored under a pseudonym (study number). You can decide at any time that you no longer wish to participate in the study.

Step 1: Are you suitable to participate?

- You may participate in the study if you:
- Are 18 years of age or older
- Are pregnant with a singleton (1 child)
- Are 32 to 37 weeks pregnant
- Are admitted in the obstetric department
- Have been asked to come to the outpatient clinic at least 2 times a week for extra check-ups for you and your child.

Unfortunately, you cannot participate in the study if:

- You have a pacemaker
- You are known to have a serious condition or skin damage that makes it impossible to stick the NRM patch
- You do not have a sufficient understanding of the Dutch or English language
- You or your unborn child are known to have a cardiac arrhythmia
- You have been admitted because of blood poisoning (sepsis)
- Your placenta is close to or over the cervix
- You have very high blood pressure for which you receive medication through an IV access
- There are serious concerns about you and/or your unborn child for which the doctor recommends that your child will be delivered soon

Step 2: the method of monitoring

Because the study investigates the feasibility of future home monitoring, this study will take place physically in the hospital at the outpatient clinic or during your admission. This way, we can safely evaluate whether it is possible for you to carry out the measurement independently. You measure the heart rate of the baby with the NRM, and the signal is sent to the hospital as if it were coming from your home. This allows us to safely evaluate whether the signals actually arrives in your file.

In this study, you therefore do the eCTG measurement yourself. In case of hospitalization, this will take place every day in your own room. If your check-ups are scheduled at the outpatient clinic, you will perform an eCTG measurement yourself during each check-up appointment in a room reserved for this purpose. Because you perform this measurement yourself, your appointment may take 5 minutes longer. Once you have completed the eCTG measurement yourself a few times, your appointment will probably be shorter, because you will not have to wait for the healthcare professional. The number of measurements depends on the number of appointments scheduled for you and will take place between 2 to 7 days a week. You will receive an explanation in advance how to perform the measurement.

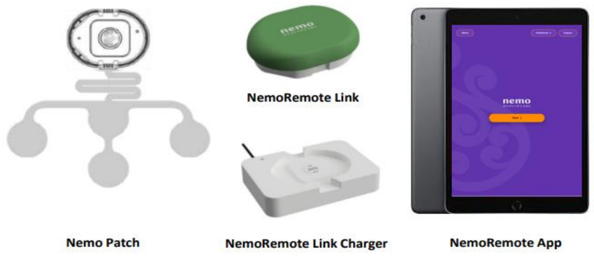

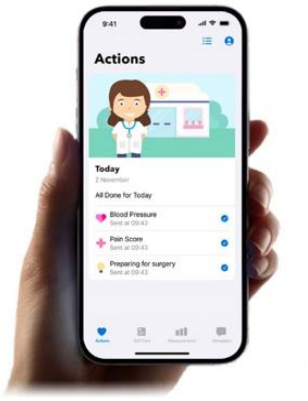

Figure 3. NRM set (eCTG) *Figure 4. Luscii® app*

Step 3: Surveys and measurements

You do not have to come to the hospital extra for the measurements. Measurements and data collection will take place during your admission to the obstetric department (you are already in the hospital) and/or during your scheduled appointments at the outpatient clinic (standard care). For an overview of the steps in this study, see Appendix C.

<u>You take the following measurements:</u>

You stick the patch every day and use it to measure your heart rate, your child's heart rate and the activity of your uterus. This will be every day in case of hospital admission, and at least 2 times a week if your check-ups are at the outpatient clinic. You also measure your blood pressure, your heart rate and your temperature yourself. You keep track of these measurements, physical complaints and other information (such as your child's movements) in the Luscii® app on your phone or tablet. Your healthcare provider will immediately see your eCTG measurement at the ward. The healthcare provider will also look at the information you have entered in the Luscii® app and note it in your file. After that, the PA or doctor will call you on the phone to discuss everything with you and to indicate whether the eCTG measurement can be completed.

Questionnaires

You will be asked to fill out a digital questionnaire 3 times. The questions concern your health, your experiences with the NRM (eCTG), and your satisfaction with the care you received. The questionnaires are sent to your email address and stored under a pseudonym (linked to a unique personal code). Your experiences are very valuable for our study and will be included in the results of the study.

<u>Questionnaire 1:</u> concerning your health while using the eCTG monitoring.

- You will receive this questionnaire on the first day after you have used the eCTG for the first time.
- Completion takes about 3-5 minutes (6 multiple choice questions + 1 question to which you answer with a score of 0 to 10)

<u>Questionnaire 2:</u> concerning your experiences with the eCTG.

- You will receive this questionnaire after you are discharged from the hospital or when you no longer need to come to the outpatient clinic for extra check-ups.
- Completing it will take you about 5-10 minutes (15 multiple-choice questions with the option to give a reason if you are not satisfied)

<u>Questionnaire 3:</u> concerning your health after the birth of your child and about how you experienced the care.

- You will receive this questionnaire 4 weeks after the birth of your child.
- Completing it will take you about 5-8 minutes (14 multiple choice questions + scale measurement from 0 to 10)

What is different from regular care?

There is not much different in this study than in standard care. The eCTG measurements in this study will replace the planned CTG measurements. The appointments scheduled with the doctor will also continue as usual. If you are admitted to the hospital, the doctor will also come to your room for the daily visit. The main difference in participating in this study is that you will perform the eCTG measurement yourself as well as the blood pressure and temperature measurements, which will make you more independent during your admission and/or at the outpatient clinic. You will receive a call from the PA or doctor. The daily visit will also take place in the room. You fill in the answers via the Luscii® app, which is already used as standard care in the Netherlands and in the MMC in other departments. In the MMC, the Luscii® pregnancy app is currently only available to participants of this study.

<u>Additional ultrasound</u>

For 24 participants in the study there is an opportunity for an extra ultrasound. This ultrasound is performed during an eCTG measurement and studies a blood vessel in the umbilical cord and a blood vessel in the uterus. The purpose of the ultrasound is to see if it is possible to perform these measurements at the same time. In addition, the measurements will be used as a first step to predict problems in pregnancy (such as high blood pressure) in the future. Your doctor will indicate whether you are eligible for this, and discuss whether you want to get this extra measurement. This ultrasound will take about 15 minutes and the researcher will visit you in your (outpatient) room. The ultrasound does not provide a different treatment plan for you. Patients who do not fall into this group will only receive the standard ultrasounds.

5. What do we expect from you?

We want the study to go well and that is why we ask you to:

- Contact the practitioner/researcher if:
    - You experience skin irritation at the site of the patch.
    - You no longer want to participate in the study. You can always decide that you no longer want to participate in the study.
    - Your phone number, address, or email address changes.
- Register your measurements, complaints and other necessary information in the Luscii app® in the way that the researcher has explained to you.
- Come to every appointment at the outpatient clinic.

6. What side effects, adverse effects or discomforts can you experience?

The NRM is approved for use in the clinic. The examination through the patch on the abdomen is a very safe method and is not harmful to you or your unborn child. In some cases, the patch can cause (mild) skin irritation. If skin irritation occurs, report it to your practitioner or the researcher. Sometimes moving the patch is sufficient, sometimes it is necessary to switch to the standard way of monitoring. The skin irritation will disappear on its own, treatment is not necessary.

7. What are the advantages and disadvantages of participating in the study?

Participating in the study can have advantages and disadvantages. We list them below. Think about this carefully, and talk about it with others.

Possible benefits of participating in the study may include:

- The NRM is wireless and gives you more freedom of movement. You are allowed to move during registration.
- The NRM ensures that you don't have to wear tight straps around your waist.
- You perform most of the measurements yourself, which makes you less dependent on the care providers.

Possible disadvantages of participating in the study may include:

- Possible (mild) skin irritation due to the patch.
- If there is insufficient signal, it may be necessary to switch to the standard method of surveillance.
- When the extra ultrasound is made for you, it will take you 15 minutes. The ultrasound will take place during your admission to the obstetric department (or at the outpatient clinic) during your eCTG measurement and will not interfere with your standard treatment.

Don't want to participate?

You decide whether to participate in the study. Participation is voluntary. Don't want to participate? Then you get the standard way of surveillance. You don't have to explain why you don't want to participate.

8. When does the research stop?

The researcher will let you know if there is any new information about the study that is important to you. The researcher will then ask you whether you will continue to participate.

In these situations, the investigation stops for you:

- All examinations according to the schedule are over.
- You want to stop participation to the study. You can decide at any time to stop participating to the study. Then report this to the researcher immediately. You don't have to tell why you are quitting. You will then receive the standard treatment again.
- The researcher thinks it is better for you to stop. The researcher will still invite you to fill in any subsequent questionnaires.
- One of the following decides that the study should stop: the MMC, the government, or the medical ethics committee that assesses the study.

What happens if you stop the study?

The researchers use the data collected up to the moment of stopping.

The entire study is over when all the participants' measurements are finished.

9. What happens after the examination?

After processing all the data, the researcher will publish the results of the study in scientific journals, among others. If you wish, we can inform you by email about the most important results of the survey.

10. What do we do with your data?

Do you participate in the study? Then you also give permission for your data to be collected, used and stored. In addition, we ask for your permission to share your pseudonymized (study number) study data with other researchers within the MMC in the context of similar research. You can indicate whether you agree on the consent form in Appendix D.

What data do we collect?

Your personal data will be used and stored for this study. This concerns data such as your name, address, date of birth and data from the delivery and data about your health until the follow-up check-up (6 weeks after your

birth). A number of data about your child are also used and stored, such as birth weight, Apgar scores, umbilical cord blood values, possible admission to the pediatric ward and general information about your child's health. Your child's outcomes will also be monitored until discharge from the hospital. If the discharge takes place within 6 weeks after birth, we will follow up on the results until then.

Why do we collect, use, store and share your data?
We collect, use, store and share your data in order to answer the questions of this study. And to be able to publish the results. This gives practitioners and researchers in the Netherlands and in the world access to our knowledge and allows further research to take place, and prevents duplication of research.

How do we protect your privacy?
To protect your privacy, we link a unique personal code to your data. We keep the key to the code in a secure place in the MMC. When we process your data, we always use only that code. Even in reports and publications about the investigation, no one can recall that it was about you.

Who can see your data?
Some people, in addition to the study team, can access the non-anonymized data at the study site (MMC), including the data without code. This is necessary to be able to check whether the examination has been carried out properly and reliably. Persons who are given access to your data for verification are: the committee that monitors the safety of the study, a monitor (this is someone who check-ups whether the rights of the subjects are properly protected, the study data is correct and reliable, and the study meets the legal requirements) hired by MMC and national supervisory authorities, for example, the Health and Youth Care Inspectorate. They keep your data confidential. We ask you to give permission for this inspection. Data of the heart rhythm of the unborn child and the contraction activity measured with the NRM® will be provided in a secure environment with Nemo Healthcare® (the company that developed the NRM) to answer the study question. Nemo Healthcare® does not have access to direct data that can be traced back to you and therefore only receives anonymized measurements.

How long do we keep your data?
We are legally obliged to store your data in the hospital (MMC, Veldhoven) for 15 years. You give permission for this when signing the consent form.

Can we use your data for other (local) research?
After this study, your data may also be important for other scientific research that we may do in the future in the field of monitoring pregnant women. By signing the consent form, you indicate that you agree to this (*Appendix D*).

You share data with other (external) researchers
In order to make it possible to publish the results of this study in certain scientific journals and to give other researchers outside the MMC the opportunity to conduct further research, it is necessary to make anonymized data available. Data will not be traced back to you in any way. You give permission for the exchange of this data.

What happens in the event of unexpected discoveries?
During the examination, we may happen to find something that is important for your health or the health of your child. The researcher will then contact your practitioner. You will then discuss with your general practitioner or

specialist what needs to be done. With the form, you give permission to inform your general practitioner or specialist.

Can you withdraw your consent to the use of your data?

You can withdraw your consent to the use of your data at any time. This applies to the use in this study and to the use in other research. But beware: do you withdraw your consent, and have researchers already collected data for a study? Then they may still use this data.

Where can you find more information about your personal data?

For general information about your rights when processing your personal data, please consult the website of the Dutch Data Protection Authority (www.autoriteitpersoonsgegevens.nl). If you have any questions or complaints about the processing of your personal data, we recommend that you first contact the researchers. You can also contact the Data Protection Officer of the institution, for contact details please refer to Appendix A, or the Data Protection Authority.

11. Will you be compensated if you participate in the study?

Participation in the study is without costs. You will not be reimbursed if you participate in this study.

12. Are you insured during the examination?

Insurance has been taken out for everyone who participates in this study. The insurance pays for damage caused by the examination. But not for all damage. In Appendix B you will find more information about the insurance and the exceptions. It also states to whom you can report damage.

13. Exchange of information

If your primary practitioner does not work in MMC, the investigator will contact your primary practitioner to request your medical information that is important for this study. If you are under the supervision of another hospital during your pregnancy or have been (briefly) admitted to MMC and then return to your own hospital, this is necessary to collect your complete data. This concerns, for example, the date of delivery, the health of your child and your health up to 6 weeks after your delivery. You consent to the exchange of this data.

14. Do you have any questions?

Questions about the examination can be asked to your practitioner on the ward or to the investigation team. Do you want advice from someone who is independent ? Then you can contact Dr. H.J. Niemarkt, pediatrician at the MMC. The contact details are in Appendix A. He knows a lot about the study, but is not part of the study team. Do you have a complaint? Then discuss this with the researcher or the doctor who treats you. Would you rather not do this? Then go to the MMC complaints officer. Appendix A explains how to contact them.

15. How do you give permission for the study?

You can first think calmly about this study. Then you tell the researcher whether you understand the information and whether or not you want to participate. Would you like to participate? Then fill out the consent form that you will find with this information letter. You and the researcher will both receive a signed version of this consent declaration form.

Thank you for your time, Team **HASTA**

16. Annexes to this information

A. Contact details

B. Overview of study interventions

C. Insurance information

D. Consent form

Appendix A: contact details for the Máxima Medical Center

Researchers:

Dr. J.O.E.H. van Laar, gynaecologist, Máxima MC Veldhoven

Dr. L. Monen, gynaecologist, Máxima MC Veldhoven

Dr. Ir. M.B. van der Hout-van der Jagt, medical engineer, Máxima MC Veldhoven

S. van Weelden, MSc, PA, researcher gynecology/obstetrics, Máxima MC Veldhoven

Phone: 040-8888384

Email: *hasta.studie@mmc.nl*, *Gynaecologie.secr@mmc.nl*

Independent expert:

Dr. H.J. Niemarkt, pediatrician-neonatologist, Máxima MC Veldhoven

Phone: 040-8889350

Email: *kindergeneeskunde@mmc.nl*

Quality and Safety Department

If you have any questions and/or comments regarding your rights and/or the processing of your personal data within Máxima MC, please contact the Data Protection Officer. Do you have a complaint about the processing of your personal data? Please contact the investigation team or the complaints officer.

Phone: (040) 8888366

Email: *gegevensbescherming@mmc.nl*

Website:

- *www.mmc.nl/privacy*
- *www.mmc.nl/over-ons/innovatie-wetenschap/beoordeling-onderzoek-metc/informatie-met- Regarding-the-General-Regulation-Data-Protection-for-Participants-in-Clinical-Research/*
- *www.autoriteitpersoonsgegevens.nl*

Complaints:

If you have any complaints, please contact the hospital complaints officer.

Phone: (040) 8889481

Email: *klachtenfunctionaris@mmc.nl*

Appendix B: overview of study interventions

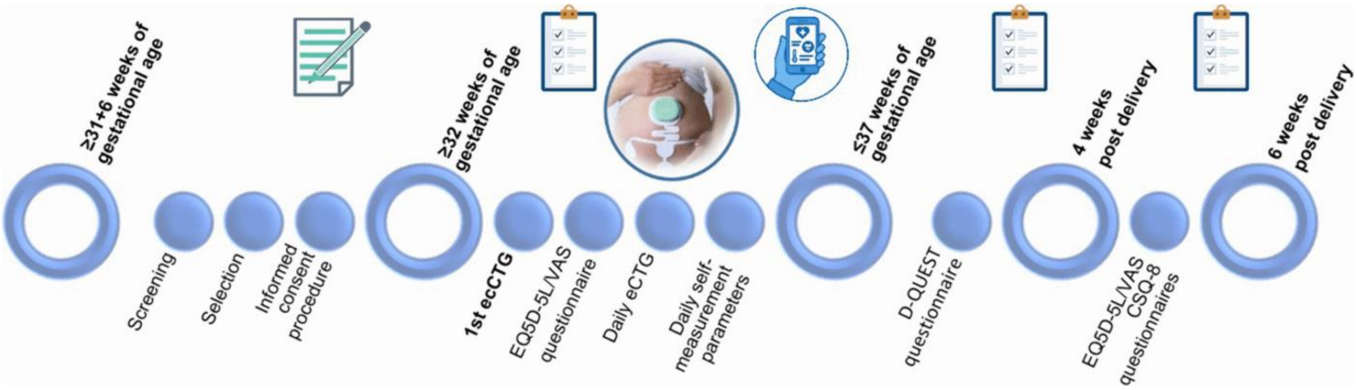

Appendix C – information about the insurance

The Máxima MC has taken out insurance for everyone who takes part in the study. The insurance pays for the damage you have suffered because you participated in the study. This concerns damage you suffer during the study or within 4 years after you participated in the study. You must report damage to the insurer within 4 years.

Have you suffered damage as a result of the study? Please report this to the researcher and the insurer: Centramed, po box 7374, 2701 AJ Zoetermeer, Phone: 070-301 7070, e-mail: *info@centramed.nl*

The insurer of the research is:<br>Name: Centramed<br>Address: PO Box 7374, 2701 AJ Zoetermeer<br>Phone number: 070-301 7070<br>E-mail: info@centramed.nl<br>Policy number: 624.100.045

The insurance pays a maximum of €650.000 per person, a maximum of €5.000.000 for the entire study, and a maximum of €7.500.000 per year for all studies by the same sponsor.

Please note that insurance does **not** cover the following damage:

- Damage due to a risk about which we have given you information in this sheet. But this does not apply if the risk turned out to be greater than we previously thought. Or if the risk was very unlikely.
- Damage to your health that would also have happened if you had not taken part in the study.
- Damage that happens because you did not follow directions or instructions or did not follow them properly.
- Damage caused by a treatment method that already exists. Or by research into a treatment method that already exists.

These provisions can be found in the 'Besluit verplichte verzekering bij medisch-wetenschappelijk onderzoek met mensen 2015' (Medical Research (Human Subjects) Compulsory Insurance Decree 2015'). This decision can be found in the Government Law Gazette (https://wetten.overheid.nl)

Appendix D: Subject consent form and consent parent(s)/legal representative(s) of the child.

Data collection of feasibility of external "home" monitoring of mother and child during pregnancy at the hospital up to six weeks after delivery.

- I have read the information letter. I could also ask questions. My questions have been answered well enough. I have had enough time to decide if I want to participate in this study.
- I know that participation is voluntary. I also know that I can decide at any time not to participate or to stop in the study without giving a reason.
- I give the researcher permission to inform my GP/specialist(s) who are treating me that I am participating in this study.
- I give permission for researchers to collect and use my personal and medical data and the data of my child from the electronic patient file or questionnaires for the purpose of answering the research question in this study.
- I give the researcher permission to provide my GP or specialist information about unexpected findings from the study that are important for my health.
- I give permission for researchers to request medical information from me and/or my child after delivery from my practitioner, primary care physician, obstetrician, gynaecologist, child health center or pediatrician to answer the study question of this study.
- I know that in order to monitor the study, some people may have access to all my data. These people are listed in this information letter. I consent to such access by these people.
- I give permission to keep my data stored for 15 years at the study location.

Please choose Yes or No in the table below

I consent to store my data to use it for <u>other research</u> (within the MMC), as stated in the information letter.	**Yes ☐**	**No ☐**
I consent to be <u>asked</u> after this study whether I would like to <u>participate</u> in a follow-up study.	**Yes ☐**	**No ☐**
I would like to be <u>informed</u> about the results of this study.	**Yes ☐**	**No ☐**

I want to participate in this study.

My name is (participant): ……………………………………………………………
Date of birth: ___ / ___ / ___

Email address: …………………………………………………………………………………

Signature: ………………………………………………… Date : ___ / ___ / ___

……

If there is another (authoritative) parent, legal representative(s) or guardian besides you (the mother), we also ask that person to give separate permission for the collection of your child's data after birth.

Is there **another (authoritative) parent, legal representative(s) or guardian** besides you?

☐ **Yes**
☐ **No**
If you answered YES, the person must sign below for permission. If that person does not give permission, we are not allowed to collect your child's data.

The name of the other (authoritative) parent, legal representative(s) or guardian is:

Name: ………………………………………………………………………………………………………..

Signature: …………………………… Date: ___ / ___ / ___

The following is completed by the treating **physician or researcher**
I declare that I have fully informed this subject study.

Will information become known during the study that could influence the subject's consent? Then I will inform her in a timely fashion.

Name researcher (or her representative):……………………………..

Signature:……………………… Date: ___ / ___ / ___

** The study subject will receive the full information sheet together with a signed copy of the consent form.*

Appendix D: Subject consent form and consent parent(s)/legal representative(s) of the child.

Data collection of feasibility of external "home" monitoring of mother and child during pregnancy at the hospital up to six weeks after delivery.

- I have read the information letter. I could also ask questions. My questions have been answered well enough. I have had enough time to decide if I want to participate in this study.
- I know that participation is voluntary. I also know that I can decide at any time not to participate or to stop in the study without giving a reason.
- I give the researcher permission to inform my GP/specialist(s) who are treating me that I am participating in this study.
- I give permission for researchers to collect and use my personal and medical data and the data of my child from the electronic patient file or questionnaires for the purpose of answering the research question in this study.
- I give the researcher permission to provide my GP or specialist with information about unexpected findings from the study that are important for my health.
- I give permission for researchers to request medical information from me and/or my child after delivery from my practitioner, primary care physician, obstetrician, gynaecologist, child health center or pediatrician to answer the study question of this study.
- I know that in order to monitor the study, some people may have access to all my data. These people are listed in this information letter. I consent to such access by these people.
- I give permission to keep my data stored for 15 years at the study location.

Please choose Yes or No in the table below

I consent to store my data to use it for <u>other research</u> (within the MMC), as stated in the information letter.	**Yes ☐**	**No ☐**
I consent to be <u>asked</u> after this study whether I would like to <u>participate</u> in a follow-up study.	**Yes ☐**	**No ☐**
I would like to be <u>informed</u> about the results of this study.	**Yes ☐**	**No ☐**

I want to participate in this study.

My name is (participant): ………………………………………………………………

Date of birth: ___ / ___ / ___

Email address: ……………………………………………………………………………………

Signature: ………………………………………………… Date : ___ / ___ / ___

………

If there is another (authoritative) parent, legal representative(s) or guardian besides you (the mother), we also ask that person to give separate permission for the collection of your child's data after birth.

Is there **another (authoritative) parent, legal representative(s) or guardian** besides you?

☐ **Yes**

☐ **No**

If you answered YES, the person must sign below for permission. If that person does not give permission, we are not allowed to collect your child's data.

The name of the other (authoritative) parent, legal representative(s) or guardian is:

Name: ……………………………………………………………………………………………………..

Signature: ……………………………   Date: ___ / ___ / ___

The following is completed by the treating **physician or researcher**
I declare that I have fully informed this subject study.

Will information become known during the study that could influence the subject's consent? Then I will inform her in a timely fashion.

Name researcher (or her representative):……………………………….

Signature:………………………   Date: ___ / ___ / ___

** The study subject will receive the full information sheet together with a signed copy of the consent form.*
